# Tuning the Crystal Structure of Amphiphilic 3,4,5-Tris(alkyloxy)benzenesulfonates with Bulky Tetrabutylammonium Cations by Variation in the Aliphatic Chain

**DOI:** 10.3390/molecules31030401

**Published:** 2026-01-23

**Authors:** Aleksei Stupnikov, Artem Bakirov, Maxim Shcherbina, Enfeng Song, Uwe Beginn, Martin Möller, Sergei Chvalun

**Affiliations:** 1 Enikolopov Institute of Synthetic Polymeric Materials, Russian Academy of Science, 70 ul. Profsoyuznaya, 117393 Moscow, Russia; bakirov.artem@gmail.com (A.B.); max-shcherbina@yandex.ru (M.S.); s-chvalun@yandex.ru (S.C.); 2 Moscow Center for Advanced Studies, 20 Kulakova Str., 123592 Moscow, Russia; 3 Organic Materials Chemistry, Institute for Chemistry, University Osnabrück, D-49069 Osnabrück, Germany; elf_song@163.com (E.S.); ubeginn@uni-osnabrueck.de (U.B.); 4 Institute of Technical and Macromolecular Chemistry, RWTH Aachen and DWI e.V., D-52056 Aachen, Germany; moeller@dwi.rwth-aachen.de

**Keywords:** self-assembly, self-organization, liquid crystals, wedge-shaped dendrons, phase behavior, octupole organization, bulky focal groups

## Abstract

3,4,5-Tris(alkyloxy)benzenesulfonates constitute a class of wedge-shaped dendrons exhibiting liquid crystalline properties, characterized by a diverse and intricate phase behavior that is predominantly governed by the length of the terminal aliphatic chains and the size and nature of the dendrons’ focal group, which collectively influence their self-organization. In systems comprising both two large counterions—wedge-shaped 3,4,5-tris(alkyloxy)benzenesulfonate anions and tetrabutylammonium (N(C_4_H_9_)_4_^+^) cations—a delicate balance between ionic interactions and microphase segregation drives the formation of giant octupoles that self-assemble subsequently into a remarkably ordered lattice. Such phase behavior is consistently observed across a broad range of compounds, with terminal aliphatic chain lengths varying from six to eighteen methylene units. While the structural configuration of the octupoles and the resulting crystalline lattice remains consistent across most compounds, a distinct transition from quantitative to qualitative changes is observed in tetrabutylammonium 3,4,5-tris(hexadecyloxy)benzenesulfonates and 3,4,5-tris(octadecyloxy)benzenesulfonates that exhibit a unique crystalline lattice structure.

## 1. Introduction

Self-organization of complex systems is a field of supramolecular chemistry that has attracted a lot of scientists, and among them, the contributions of Haken [1], Prigozhin [2], Bak [3], Samarsky and Kurdumov [4] should be mentioned. Their input has led to a deep understanding of such fundamental property of Mother Nature as self-organization that manifests itself everywhere–from large galactic clusters to the evolution of the Life on our planet.

At present, the phenomena of self-assembling and self-organization have been and are being studied for a wide range of synthetic and biological amphiphilic molecules [5,6,7]. Wedge-shaped dendrons based on benzenesulfonic acid have proven themselves to be prospective self-organizing units for designing functional materials [8]. It was revealed that such dendrons, depending on the architecture of the mesogenic group [9,10], size of the focal group [11,12], aliphatic chain attachment to the mesogenic group [13], and their length [14] can form one-dimensional—smectic, two-dimensional—columnar hexagonal, and three-dimensional—cubic, bicontinuous mesophases [15,16]. Up until now, the ability to make rather precise predictions on the phase behavior of a particular material with wedge-shaped molecules and on the structure of the formed supramolecular aggregates has made it possible to create scalable ion-selective membranes with variable ion channel size [17,18,19,20,21]. Moreover, it is possible to design ionic liquid crystals that can respond to external stimuli such as light [22,23,24,25,26], chemical compounds [27,28,29,30], mechanical pressure [31], electrical field [32], and humidity [33].

For instance, variation in dendron shape by changing the length of aliphatic chains from six to twelve methylene units in cesium 3,4,5-tris(alkyloxy)benzenesulfonates leads to an increase in the diameter of the central ion channel in the cylindrical supramolecular aggregates of ordered hexagonal columnar mesophase, providing a possibility of fabricating selective ion channel membranes [34]. The main parameter that determines the shape of forming supramolecular aggregates, and hence, the type of stable mesophase, is the equilibrium average interface curvature [35,36,37]. In wedge-shaped dendrons, variation in the length of aliphatic chains serves a dual purpose. Apart from defining the aforementioned amphiphilic balance, it also determines the solid angle of the dendrons.

On the other hand, changing the focal group size has a significant influence on the phase behavior of such systems, as reported for alkali 2,3,4-tris(dodecyloxy) benzenesulfonates earlier [12]. It was revealed that the formation of a cubic phase is characteristic of small ions, while their increase leads to the stability enhancement of columnar mesophase. Moreover, it has been shown that increasing the size of the inorganic counter ion strongly affects the columnar phase aggregate diameter, but also increases the transition temperature to plastic crystal.

Comparative analysis of phase behavior of benzenesulfonic salts [38] revealed that the increase in the counter ion size from lithium to cesium and tetramethylammonium in 2,3,4-tris(dodecyloxy)benzenesulfonates leads to a more tapered molecular shape and favors the formation of columnar phases. It should be noted that higher ionic strength increases the temperature range stability of the columnar phase. One can quantify this argumentation by introducing the shape parameter:

$$P=\frac{V}{A\cdot l_{C}}$$

where *V* is the volume of aliphatic chains, *l*_c_ is their length, and *A* is the cross-section of the focal group. Such parameter changes in the range from 25 (lithium) to 4.7 (cesium) for alkali salts of 3,4,5-tris (octadecyloxy) benzenesulfonic acid (*l* = 18 carbon atoms), as the wedge-angle of the dendron. Thus, the preferred modus of self-assembly for lithium benzenesulfonates is the formation of spherical micelles organizing cubic plastic crystals, while sodium, potassium, and cesium benzenesulfonates form columnar mesophases.

One should note that X-Ray studies of such materials represent a number of particular difficulties that are inextricably linked with the nature of soft matter and its partially ordered systems. First, scattering patterns of such materials are characterized by a relatively small number of reflections [39,40,41]; thus, the solution of the inverse scattering problem—definition of the structure of the formed supramolecular aggregates—is limited by a comparatively low resolution [42,43]. Second, even the solution of the direct scattering problem—the calculation of the scattering pattern by a partially ordered mesophase—is not so obvious. Such mesophases usually contain more or less ordered supramolecular aggregates that are immersed in a substantially less ordered matrix (in 2,3,4- and 3,4,5-*tris*(dodecyloxy)benzenesulfonates, the matrix is represented by the regions of segregated aliphatic chains) [44]. How should this matrix be interpreted on calculation of the diffraction patterns? Taking them into account leads to the fixation of several mutual distances between different aliphatic atoms and between aliphatic and mesogen atoms, which, in turn, define a large number of surrogate calculated reflections (although in reality they do not exist as the matrix is disordered) that flood the calculated diffraction pattern. Another way is to ignore aliphatic reflections totally, and consider supramolecular aggregates as existing without aliphatic chains (“in vacuo”) [45]. Although such an approach leaves only really observed reflections, it distorts substantially intensity relationships between them, as an electron density contrast on calculation (mesogen–vacuum) drastically differs from that in reality (mesogen–aliphatics).

Such an intermediate status of the 3,4,5-tris(alkyloxy) benzenesulfonates is also observed in the studies of Low-Molecular-Weight Organogels (LMOGs). Organogels are defined as “elastic- or viscoelastic materials consisting of solvents and low molecular-mass organic gelators” [46,47]. In a solvent, the LMOG molecules can spontaneously self-assemble by means of non-covalent physical interactions such as hydrogen-bonding, solvophobic effects, van der Waals forces, π–π stacking, complexation, and electrostatic forces, to form two- or three-dimensional assemblies [48,49,50,51]. Our previous research has shown that the formation of organogels is intrinsically linked to the ability of the material to form supramolecular aggregates in bulk [52].

Thus, the majority of 3,4,5-tris(alkyloxy) benzenesulfonates with different cation counterions do form LMOGs. However, tetrabutylammonium 3,4,5-tris(alkyloxy) benzenesulfonates do not do that.

From theoretical point of view, aggregation of tetrabutylammonium 3,4,5-tris(alkyloxy) benzenesulfonates represents an important intermediate case between self-assembling of wedge-shaped and rod-like ionic crystals as diameters of bulky tetrabutylammonium and3,4,5-tris(alkyloxy) benzenesulfonic are relatively similar, their shape parameter being close to one, while an aspect ratio of their cylindrical envelope ranges from 1.8 (short aliphatic chains with the length *n* of six carbon atoms) to 3.25 (*n* = 18).

We carried out a comparative analysis of phase behavior and structure of supramolecular aggregates formed by tetrabutylammonium 3,4,5-tris(alkyloxy)benzenesulfonates with the length of the side aliphatic chain varied from six to eighteen methylene by means of small-angle and wide-angle x-ray diffraction, polarizing optical microscopy, differential scanning calorimetry, and molecular modeling.

## 2. Results and Discussion

**Figure 1:**
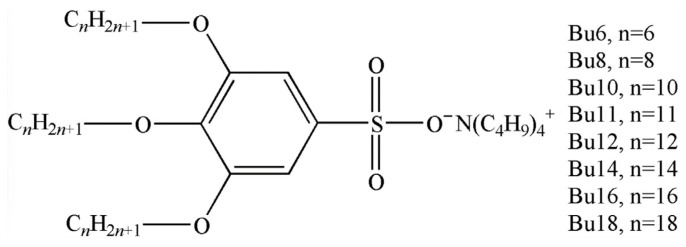
Structure of tetrabutylammonium salts of 3,4,5-tris(alkyloxy) benzenesulfonic acid.

Eight tetrabutylammonium 3,4,5-tris(alkyloxy)benzenesulfonates with different lengths of aliphatic tails were investigated, denoted hereinafter as presented in Figure 1. Synthesis of compounds **Bu6**–**Bu18** is described elsewhere [53,54].

**Figure 2:**
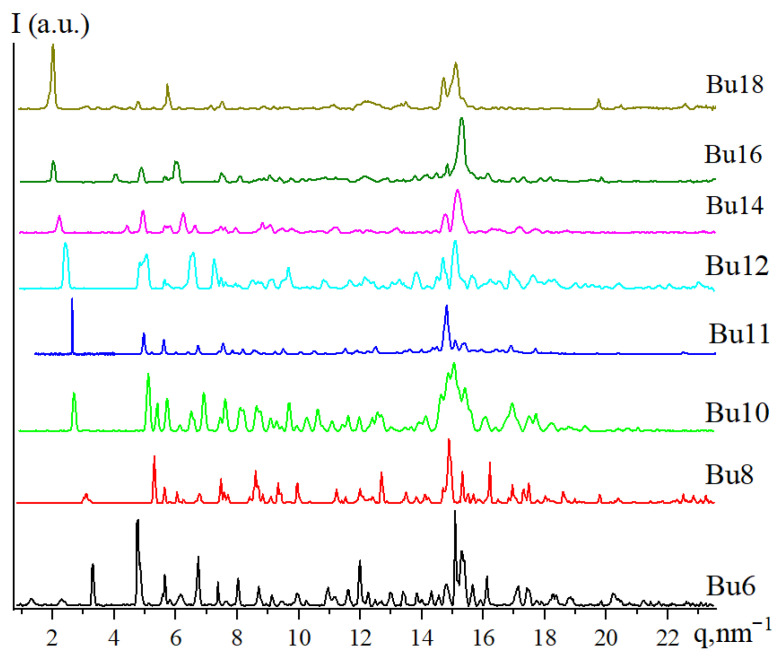
X-Ray diffractograms of compounds **Bu6**–**Bu18** at room temperature.

As it was mentioned in the Section 1, salts of 2,3,4- and 3,4,5-tris(alkyloxy) benzenesulfonic acid with small and comparatively large counterions are characterized by rich phase behavior. In contrast, DSC analysis of all the studied tetrabutylammonium 3,4,5-tris(alkyloxy)benzenesulfonates **Bu6**–**Bu18** reveals one reversible endothermic peak upon heating. Its heat of fusion is quite high (see Table 1), being more characteristic of crystal phases rather than the liquid crystal ones. Indeed, X-ray diffraction patterns obtained at room temperature for as-received samples (Figure 2) contain a significant number of narrow crystallographic peaks, clearly pointing to the crystalline order in all materials. We note also that on all X-Ray diffractograms we observed a pair of reflections characteristic of the orthorhombic packing of pure aliphatic compounds [55]—110 (*q* = 15.3 nm^−1^) and 200 (*q* = 16.9 nm^−1^). In fact, the former one (110) is among the strongest. So, one should be assured that all the tetrabutylammonium 3,4,5-tris (alkyloxy) benzenesulfonates are rather crystalline.

**Figure 3:**
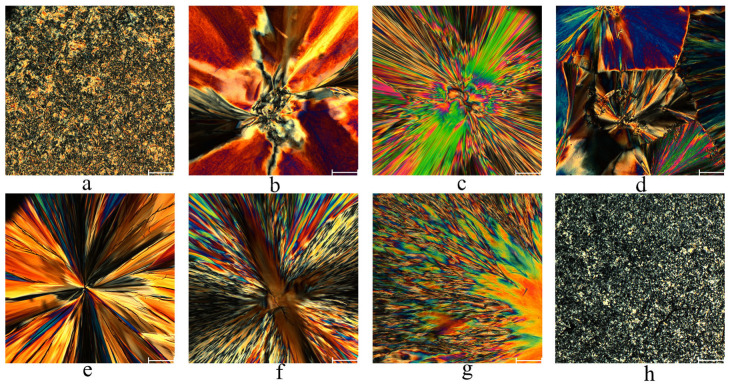
Polarizing optical microscopy images at room temperature for **Bu6**—(**a**), **Bu8**—(**b**), **Bu10**—(**c**), **Bu11**—(**d**), **Bu12**—(**e**), **Bu14**—(**f**), **Bu16**—(**g**), and **Bu18**—(**h**) (white bar’s length is 100 µm).

The most intense wide-angle peaks are typical for the crystal packing of alkanes. These conclusions are also confirmed by polarizing optical microscopy (see Figure 3). Large submillimeter domains with characteristic intensity modulations of transmitted light in the shape of a Maltese cross are present on all obtained POM microphotographs. Such patterns, as in Figure 3b–g, are characteristic of a spherulitic growth of small crystallites with correlated orientations of the crystallographic axes.

Moreover, detailed analysis of the X-ray diffractograms of compounds **Bu6**–**Bu18** reveals their substantial similarity. This is not surprising, as two subsequent compounds differ from each other by no more than just two additional methylene units in the aliphatic tails. Such a minor change in the structure of the dendron is unlikely to cause a significant deviation of the crystal cell or the shape of the supramolecular aggregate. For each compound, we performed indexing of the crystal structure; the results are presented in Table 1.

**Figure 4:**
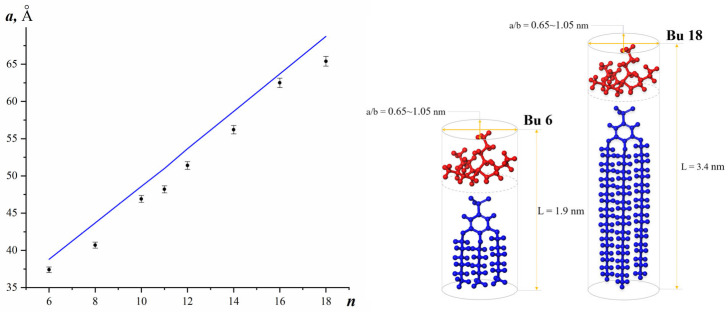
Parameter *a* of crystal lattices of 3,4,5-tris (alkyloxy) benzenesulfonates vs. chain lengths of their aliphatic tails (on the **left**). The solid line represents the calculated doubled molecular length of dendrons with extended tails by molecular modelling. Schematic of molecular dimensions for **Bu6** and **Bu18** (tetrabutylammonium cations are colored in red and benzenesulfonic derivatives colored in blue) with aspect ratios equal to 1.8 and 3.25 (on the **right**).

Compounds **Bu6**–**Bu14** form a monoclinic crystal lattice of P2, in which parameter *a* incrementally increases with the length of aliphatic tails (Figure 4), being roughly equal to the doubled molecular length. Monoclinic angle is not very large, varying from material to material in the range of 93 ÷ 97°.

Therefore, we suggest that the long axes of the dendrons are oriented along vector *a* of the crystal lattice. In compounds **Bu16** and **Bu18**, parameter *a* also correlates to the doubled molecular length and equals 62.5 and 65.4 Å, respectively. However, the cell type changes to an orthorhombic P222 one.

According to the measured macroscopic density of the samples, the number of molecules per cell was determined to be eight molecules per unit cell in all studied compounds. The number of molecules per unit cell was calculated taking into account the measured density of benzenesulfonates *ρ* (1.1 g/cm^3^), molecular mass *MM*, and the corresponding crystal cell volume *V* (see Table 1).

$$N=\frac{V\;*\;Na\;*\;\rho }{MM}$$

**Figure 5:**
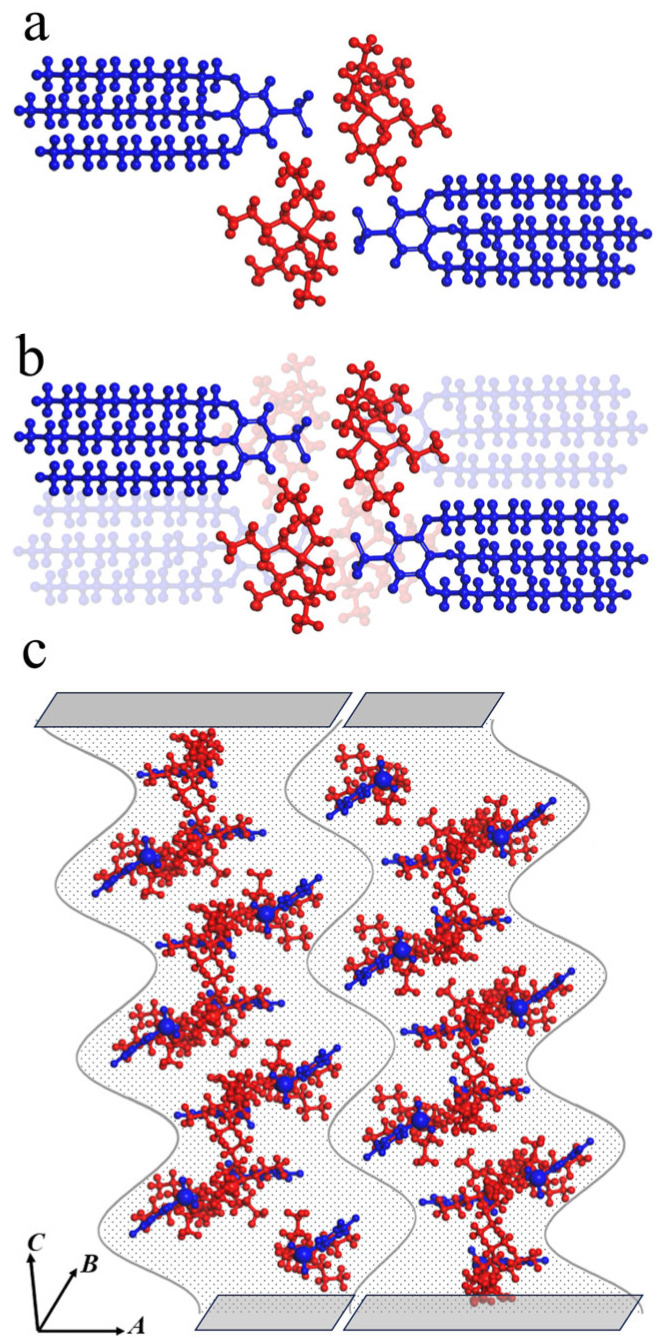
Model of molecular arrangement in monoclinic P2 cell for compound **Bu11**; cations are colored in red, anions are colored in blue, (**a**)—quadrupole, (**b**)—octupole, and (**c**)—octupole ion organization in monoclinic P2 crystal structure (supercell consists of 1×1×3 (**a**–**c**) unit cells (ions are displayed as balls and sticks, and aliphatic chains are shown as dot-filled regions for better visualization)).

The refinement of all lattices included in Table 1 revealed that their basic unit is an octupole formed by four anion-cation pairs (see Figure 5a for a particular case of the self-organization of compound **Bu11**). It is well known that the phase behavior of congruent mesogenic cation and anion pairs is determined by a delicate balance between their symmetry [56], as was shown for guanidinium and imidazolium sulphonates. Octupole organizations are indeed not that commonly observed; however, they do take place in liquid crystalline self-assembling systems [57], and π-conjugation indeed plays a significant role in such system formation.

Pawley refinement results are presented in the Supporting Information, Figure S2. Figure 5 represents a top view of the b–c plane of a quadrupole (a) and octupole (b), sulfonic cations are colored in red, mesogenic anions are colored in blue. Such an octupole consists of two quadrupole planes, each one including face-to-face anion–cation pairs (marked by blue and red colors, respectively). *P*2 symmetry of the unit cell suggests that it includes two octupoles. Octupoles are stacked along the c–axis thus forming a columnar arrangement (Figure 5c: aliphatic endings are replaced by a dot-filled region for better visualization).

The formation of octupole structures was also confirmed by molecular modeling (see Figure S5 and its discussion in the Supporting Information). We carried out optimization of the structure of ionic pairs by minimizing their free energy. On the next step, we studied, using the aforementioned Pawley refinement results, the possible mutual arrangement of the ionic pairs into quadrupoles and octupoles. It was found that the free energy of the system falls drastically on octupole motif formation. One can see the general trend towards the slight decrease in free energy in the row ionic pairs – quadrupoles – octupoles However, the most substantial input to that decrease is due to the minimization of the electrostatic part of energy (4.7 kcal/mol for dipole ionic pairs, 0.4 kcal/mol for quadrupoles, and −6.3 kcal/mol for octupoles). Such an organization is determined by two equally important factors—the aforementioned electrostatic aggregation of the ionic pairs into the octupole structures and the segregation of polar ionic and nonpolar aliphatic groups. It is already an established knowledge that 2,3,4- and 3,4,5-tris(alkyloxy) benzenesulfonic wedge-shaped dendrons form ionic channels whose structure depends on the particular shape of an ionic pair [38,58]. Figure 5b demonstrates that octupoles are also examples of such (though short) channels. However, neighboring octupoles in two consecutive (along the *c*-axis) crystal cells are shifted in the ab-plane (Figure 5c), preventing the formation of the propagating ionic channel. Thus, one can state that crystal structure formed by amphiphilic 3,4,5-tris(alkyloxy)benzenesulfonates with bulky tetrabutylammonium cations can be interpreted as columnar structure disrupted by cross-section size undulations caused by the segregation of polar and aliphatic parts of dendrons: the stability of the cylinder is determined, on the one hand, by the rigidity of the network of covalent and non-covalent bonds inside, and on the other, by the surface tension forces at its boundary, i.e., by the interaction between the aromatic mesogenic groups and the paraffin matrix of the aliphatic groups. According to Rayleigh’s theorem [59], when a liquid passes from a cylindrical jet to a round drop, the magnitude of the surface energy decreases if the height of the cylinder is greater than the circumference of its base. Therefore, the stability of a homogeneous cylinder decreases with decreasing radius—periodic disturbances of the column surface with a wavelength greater than the perimeter of the cross-section will increase until the cylinder is completely destroyed. It has been shown that elastic tensile and shear deformations also have a significant influence on the behavior of polymer cylindrical gels and the development of surface modulations in them [60]. Hardening of the cylinder surface may even lead to the formation of three-dimensionally ordered bubble structures, which we discovered for the self-assembly of gallic acid derivatives with partially fluorinated aliphatic tails [15]. Such a mechanism was theoretically explained by Leibler [35] for the columns with particularly rigid cylinder cores. Evidently, massive tetrabutylammonium counterions forming large octupole aggregates define the rigidity of the aforementioned columnar structures and their subsequent disruption.

However, Figure 5c represents the possibility of another organization of the elongated slit-like pores—along axis b between the two planes of octupoles. They are organized completely differently compared with those in cylindrical supramolecular aggregates of cesium 3,4,5-tris(alkyloxy) benzenesulfonates. The walls of such slit-like pores are formed by dendron planes that are parallel to the direction of the pore, while in cesium 3,4,5-tris(alkyloxy) benzenesulfonates, they are perpendicular to the axis of the pore.

In additional support of our conclusion, we should note that parameter b of the crystal lattice (along ionic channels) of all studied compounds is equal to 14 ÷ 15 Å, which is equal to the doubled thickness of the flat quadrupole, and corresponds, in turn, to the doubled thickness of the layer in columnar supramolecular aggregates formed by the alkali salts of 3,4,5-tris(alkyloxy) benzenesulfonic acid.

**Figure 6:**
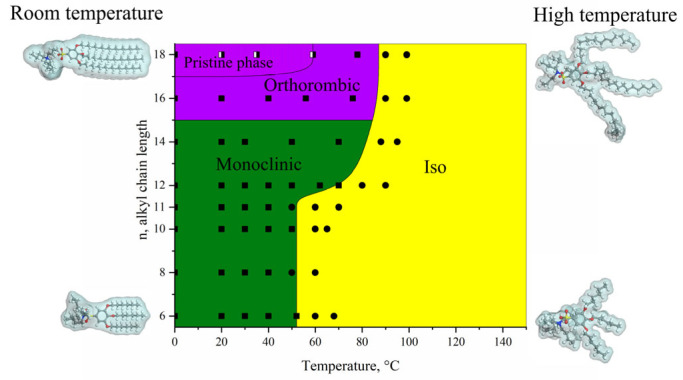
Phase diagram of tetrabutylammonium 3,4,5-tris(alkyloxy)benzenesulfonates.

Summarizing the obtained results, we can state that, in contrast to benzenesulfonates with a smaller counterion size in the focal group, tetrabutylammonium benzenesulfonates display a less complicated phase behavior and form only crystal phases of monoclinic and orthorhombic symmetry at room temperature that melt into an isotropic melt at T_max_ = 80 °C (phase diagram presented in Figure 6).

These features are closely related to the size of the bulky tetrabutylammonium ion, which is comparable to the size of the benzenesulfonic group; thus, the general shape of the (tetrabutylammonium 3,4,5-tris(alkyloxy)benzenesulfonate) ionic pair is rod-like, not tapered as for cesium benzenesulfonates. It should be noted that, analogous to benzenesulfonates with a smaller focal counter ion, the stability of phases increases with longer aliphatic chain length. However, the length of aliphatic terminal groups does not affect the molecular arrangement in the crystal cell that is governed by the octupole formation.

## 3. Materials and Methods

### 3.1. X-Ray Scattering

The structure of compounds **Bu6**–**Bu18** was investigated by small- and wide-angle X-Ray diffraction methods. SAXS and WAXS experiments were carried out at the DIKSI station of Kurchatov synchrotron (National Research Center “Kurchatov Institute”) using X-Ray radiation of 8.6 keV (1.625 Å, resolution *dE*/*E* = 10^−3^), photon flux being equal to 10^−9^ s^−1^. The beam spot on the samples was 0.3 × 0.5 mm^2^. The samples represented the fine powder with the average grain diameters equal to approximately 500 μm—the observation is supported by POM data represented in Figure 3. As the capillary diameter was 2 mm, the polycrystallinity condition for diffraction patterns was met in excess. The latter were recorded by a Dectris Pilatus (Aargau, Switzerland) 1M detector. The sample-to-detector distance was approximately 170 mm, and silver behenate was used as a standard. Data integration and processing of the raw data were performed by the Fit2D software version 18.002.

Indexing of the crystal patterns was performed using the DICVOL91 [61] program that employs the successive dichotomy method for indexing powder patterns. The powder refinement tool [62] uses a variant of the Pawley method to perform a peak shape, background, and lattice parameter refinement for an experimental powder pattern of an unknown crystal structure.

### 3.2. Thermal Analysis

Thermal analysis was carried out using the “Perkin Elmer DSC–7”(Perkin, Pleasant Plains, IL, USA) calorimeter, heating-cooling rates being 10 °C/min, respectively. Phase transition temperatures were defined as the maxima of the corresponding endo- and exothermal peaks appearing in the thermograms.

### 3.3. Polarizing Optical Microscopy (POM)

LC textures in a crossed polarizers regime were studied using the Carl Zeiss Axio Imager. M2m optical microscope (Oberkochen, Germany). The substances were placed between glass coverslips. Heating and cooling at rates of 1 ÷ 5 °C/min were carried out in a Linkam THMS600 thermal cell (Linkam, Redhill, UK). A magnifying lens with 10× magnification was used. Image processing was carried out in the Axio Vision program (v.4.8).

### 3.4. Molecular Modeling

The Biovia Materials Studio program 2023 (v.23.1.0.3829, San Diego, CA, USA) package was employed for molecular modeling of the compounds studied. We used two sets of potentials, which allow us to take into account the non-covalent interactions of the mesogenic groups inside the cylinders of the columnar mesophases: COMPASS (Condensed-phase Optimized Molecular Potentials for Atomistic Simulation Studies) and UFF (Universal Force Field). The COMPASS set is suitable for modeling of isolated molecules and condensed phases of mainly organic, polymeric, and some inorganic compounds [63,64,65]. It also allows parameterizing partial charges and valences ab initio with subsequent system optimization. To prove the results of modeling, we applied UFF potentials, used for the calculation of the geometry of organic molecules containing metal–organic complexes. UFF does not have any limitation on the chemistry of compounds involved [66,67,68].

### 3.5. Structure Confirmation, Melting Points, and Purity

**Figure 7:**
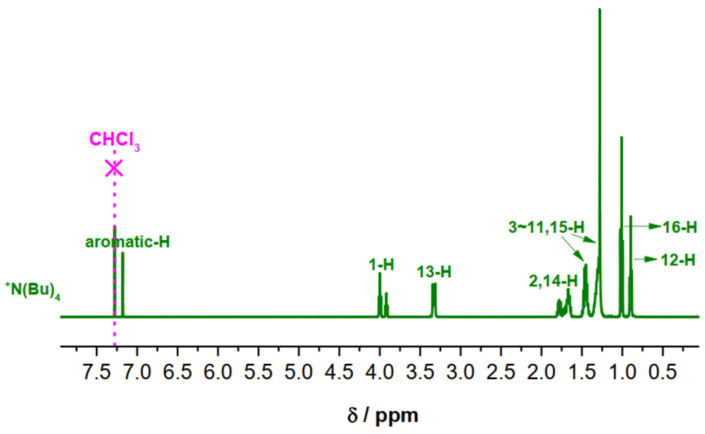
Typical 1H-NMR spectra of the monovalent cationic sulfonates (Bu)4^+^ with saturated -C_12_H_25_ chains (solvent: CDCl_3_).

The structures of the prepared compounds have been confirmed by means of ^1^H-NMR, ^13^C-NMR, and IR spectroscopy (see Supporting Information). Figure 7 depicts the ^1^H-NMR spectra of the dodecyloxy derivative **Bu12**.

All compounds exhibit a singlet of the aromatic protons around 7.25 ppm (2 protons), a multiplet at 3.95 ppm (6 protons) originating from the -CH_2_-O- groups as well as the methylene signals from the alkoxy chains between 1.4 and 1.7 ppm (60 protons), and the methyl-groups signal at 0.9 ppm (9 protons). The tetraalkylammonium salts gave rise to additional signals at 3.2–3.4 ppm (N^+^-(CH_2_)-, **Bu12**, with 8 protons). The assignment of the signals to the respective structure elements is included in Figure 1. The signals of the ^13^C-NMR spectra of all sulfonates are listed in the Supporting Information, and they confirm the structures of the molecules. The IR spectra of the salts are characterized by bands at 1185–1312 cm^−1^, implying the presence of -SO_3_^−^ groups, distinctly different from the wave numbers of the -SO_3_H vibration of the sulfonate acid educt. All IR spectra exhibit a broad absorbance at 3400–3500 cm^−1^ that must be attributed to crystal water within the sulfonates. For further characterization, all compounds were freeze-dried from benzene and stored under N_2_ prior to use. All of the synthesized compounds were checked by thin-layer chromatography (TLC) using at least two different solvent mixtures of the mobile phase to exhibit a single spot only.

According to CHNS elemental analysis, the dried compounds exceed at least 98% in purity; however, all substances contain small quantities of water (0.1–2.0 wt%) that could not be removed with freeze-drying or annealing at elevated temperatures in vacuum.

## 4. Conclusions

The ability of tapered dendrons based on symmetric and asymmetric benzenesulfonic acid for self-assembly and self-organization into a wide range of 3D (cubic), 2D (columnar), and 1D (smectic) mesophases is firmly established today. One of the most important features of such dendrons is their geometrical tapered shape that can be adjusted by variation in counterion size, chain length, and 2,3,4- or 3,4,5- attachment of aliphatic tails. In this paper, we tested the limits of such ability by a substantial change in dendron shape by the introduction of bulky tetrabutylammonium counterion that stipulates a rod-like, not-tapered habitus of the molecule. Indeed, such modification results in drastically different phase behavior of the material that tends to form crystalline structures instead of supramolecular mesophases.

The most important novelty of this manuscript is the possibility for 3,4,5-tris (alkyloxy) bezenesulfonic dendrons to adopt a completely different mode of self-assembly—not into elongated cylinders, but to the compact octupoles formed by four ionic pairs. What is the reason for such a game-changer? Is it due to excessively large cations with a shape parameter *P* roughly equal to unity? Our previous studies of self-assembly of benzenesulfonates with very long aromatic focal groups revealed the formation of smectic layers of different types [38]. It is, then, interesting to carry out the research of the ionic pairs with a little smaller, but still large, cations (e.g., tetraethylammonium or tetramethylammonium, their shape parameters being 2.0 and 4.5, correspondingly) to establish the lower limit of such a self-assembly mode—the shape parameter of cesium benzenesulfonates ranges from 4.7 (short aliphatic chains with the length *n* of six carbon atoms) to 5.1 (*n* = 18). This will certainly be the new direction of our studies.

The second important, rather theoretical, conclusion is that we have found the upper limit for the term “tapered dendron”—tetrabutylammonium 3,4,5-tris (alkyloxy) bezenesulfonates are not tapered. They do not self-assemble into cylindrical supramolecular aggregates and, consequently, do not self-organize columnar mesophase. This argument is supported by our earlier observation of the organogels, formed by the solutions of the aforementioned benzenesulfonates in different organic solvents [52]. It was revealed that, in contrast to other salts with smaller cations, tetrabutylammonium 3,4,5-tris (alkyloxy) bezenesulfonates do not form gels at all. Now we have the support for such behavior—the gels are not formed because the long supramolecular aggregates are not organized.

Certainly, the obtained results supplement the understanding of complex systems based on tapered dendron self-organization and can help to design such dendrons for specific applications.

## Figures and Tables

**Table 1:** Crystal parameters of tetrabutylammonium 3,4,5-tris(alkyloxy)benzenesulfonates.

Sample	Bu6	Bu8	Bu10	Bu11	Bu12	Bu14	Bu16	Bu18
Crystal system	Monoclinic P2	Monoclinic P2	Monoclinic P2	Monoclinic P2	Monoclinic P2	Monoclinic P2	Orthorombic P222	Orthorombic * P222
a, Å	37.4	40.7	46.9	48.2	51.4	56.2	62.4	63.7
b, Å	17.2	14.7	14.5	14.9	14.1	13.9	14.5	15.2
c, Å	13.1	15	17	17.1	16.7	16.7	14.2	14.5
β, deg	96	92	96	97	95	93	90	90
Cell V, Å^3^, E4	0.83	0.9	1.14	1.21	1.2	1.29	1.29	1.41
Isotropization temperature, °C	61	46	63	50	75	82	86	86
ΔH, J/g	34.3	32.5	53.7	46.5	76.8	71.9	76.2	50.7
Rwp (%)	16.97	18.76	6.6	10.62	17.9	8.88	15.79	18.16
Rp (%)	19.84	24.77	6.06	11.17	17.86	9.45	21.29	26.48
Observed reflections	78	102	135	101	104	130	58	21

* Annealed phase is presented in this table; pristine phase is not reproduced upon cooling and is characterized in Supporting Information, Figure S3.

## Data Availability

The data supporting this article have been included as part of the Supplementary Information.

## Supplementary Materials

The following supporting information can be downloaded at: https://www.mdpi.com/article/10.3390/molecules31030401/s1.
