# Supplementary material for: Tuning the Crystal Structure of Amphiphilic 3,4,5-Tris(alkyloxy)benzenesulfonates with Bulky Tetrabutylammonium Cations by Variation in the Aliphatic Chain
Source: Molecules. 2026 Jan 23;31(3):401. doi: 10.3390/molecules31030401 (PMC12898579; doi:10.3390/molecules31030401)
Supplement: Supplementary file 1 [file molecules-31-00401-s001.zip › molecules-4055320-supplementary.pdf]

molecules

MDPI

Article

Tuning the crystal structure of amphiphilic 3,4,5-tris(alkyloxy)benzenesulfonates with bulky tetrabutylammonium cations by variation in the aliphatic chain

Aleksei Stupnikov [1,*], Artem Bakirov [1], Maxim Shcherbina [1,2], Enfeng Song [3], Uwe Beginn [3], Martin Möller [4] and Sergei Chvalun [1]

1 Enikolopov Institute of Synthetic Polymeric Materials, Russian Academy of Science, 70 ul. Profsoyuznaya, 117393 Moscow, Russia; bakirov.artem@gmail.com (B.A.V.); max-shcherbina@yandex.ru (S.M.A.); s-chvalun@yandex.ru (C.S.N.)
2 Moscow Center for Advanced Studies, 20 Kulakova Str., 123592 Moscow, Russia
3 Organic Materials Chemistry, Institute for Chemistry, University Osnabrück, D-49069 Osnabrück, Germany; elf_song@163.com (S.E.); ubeginn@uni-osnabrueck.de (B.U.)
4 Institute of Technical and Macromolecular Chemistry, RWTH Aachen and DWI e.V., D 52056 Aachen, Germany; moeller@dwi.rwth-aachen.de
* Correspondence: alexei.stupnikov@mail.ru

Supporting information

Figure S1. DSC thermograms for tetrabutylammonium 3,4,5-tris(alkyloxy)benzenesulfonates, first heating (a) and cooling (b)

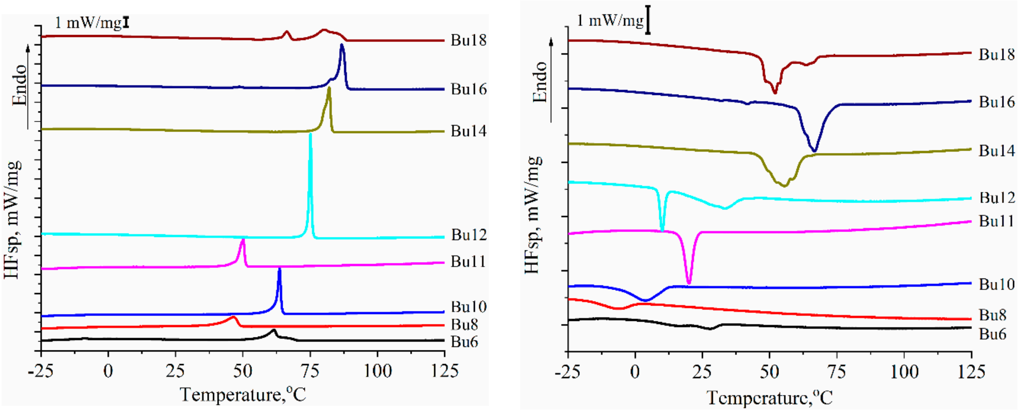

a b

Academic Editor: Marco Milanesio

Received: 4 December 2025
Revised: 19 January 2026
Accepted: 20 January 2026
Published: 23 January 2026

Copyright: © 2026 by the authors. Licensee MDPI, Basel, Switzerland. This article is an open access article distributed under the terms and conditions of the Creative Commons Attribution (CC BY) license.

Figure S2. Pawley refinement of compounds Bu6 – Bu18

Bu6	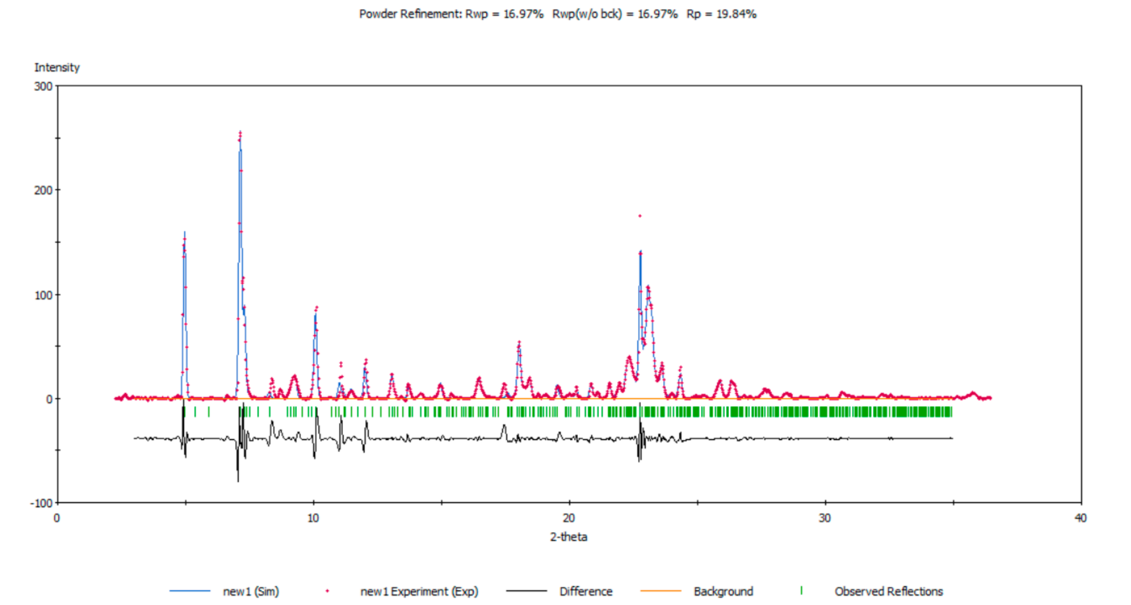
Bu8	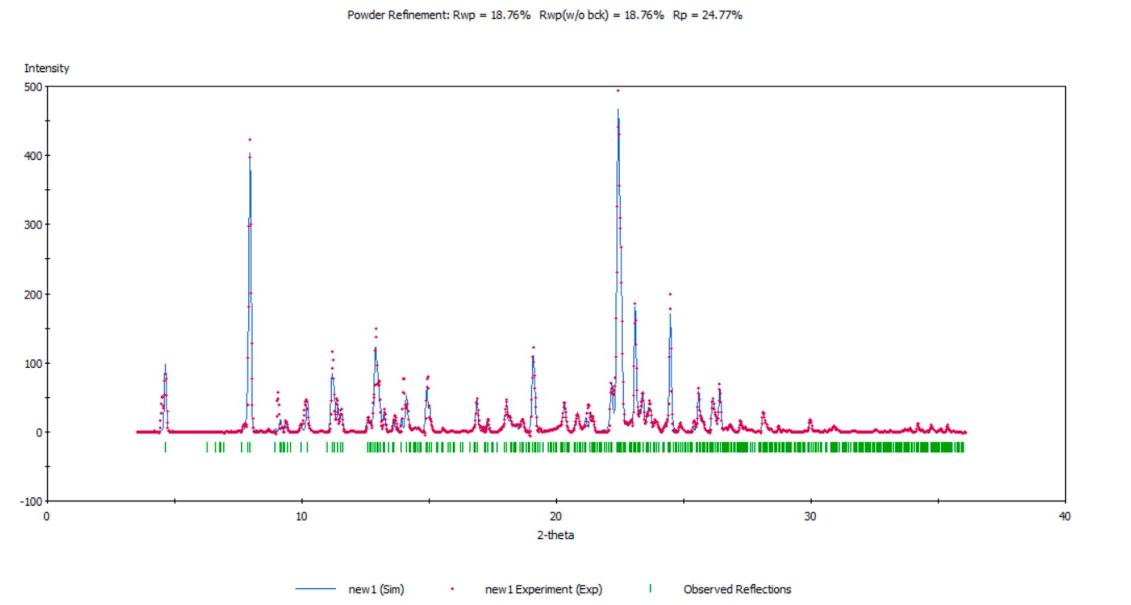
Bu10	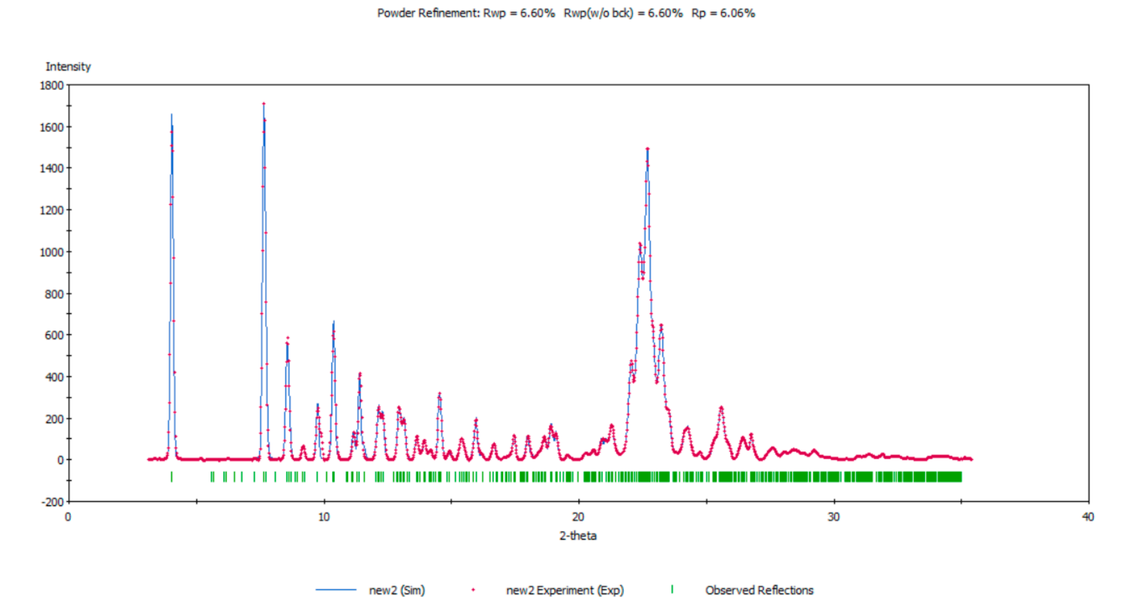

<table>
<tr><td>Bu11</td><td>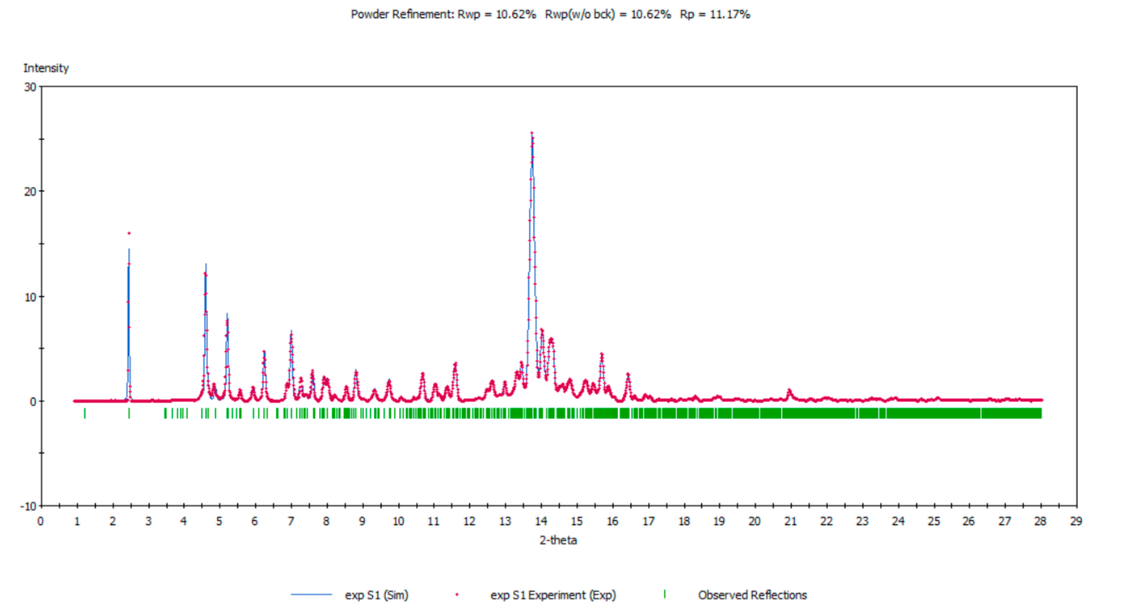
</td></tr>
<tr><td>Bu12</td><td>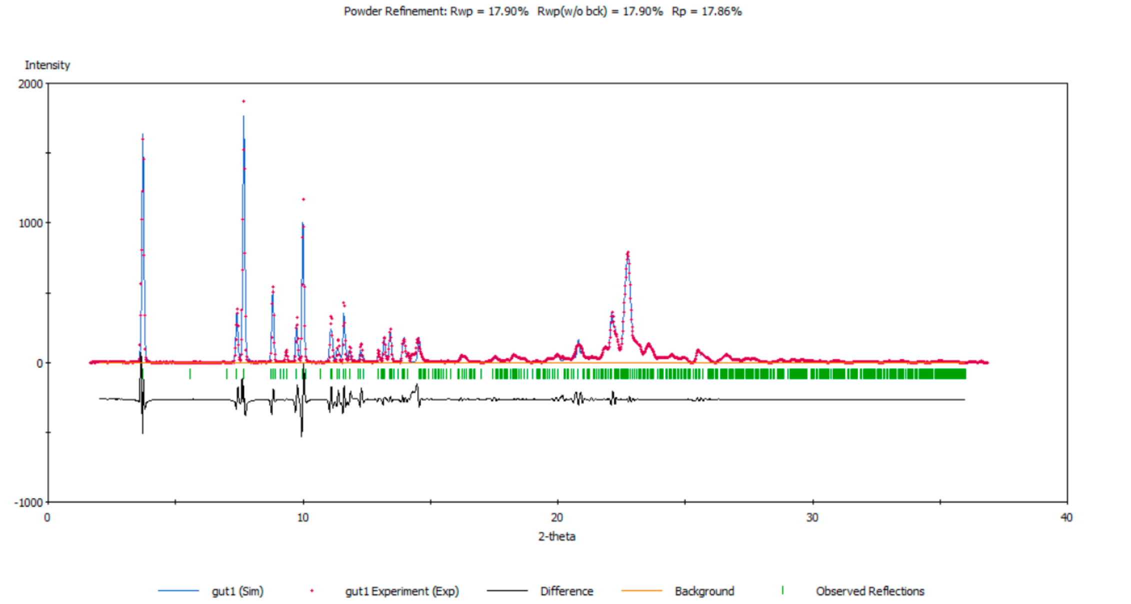
</td></tr>
<tr><td>Bu14</td><td>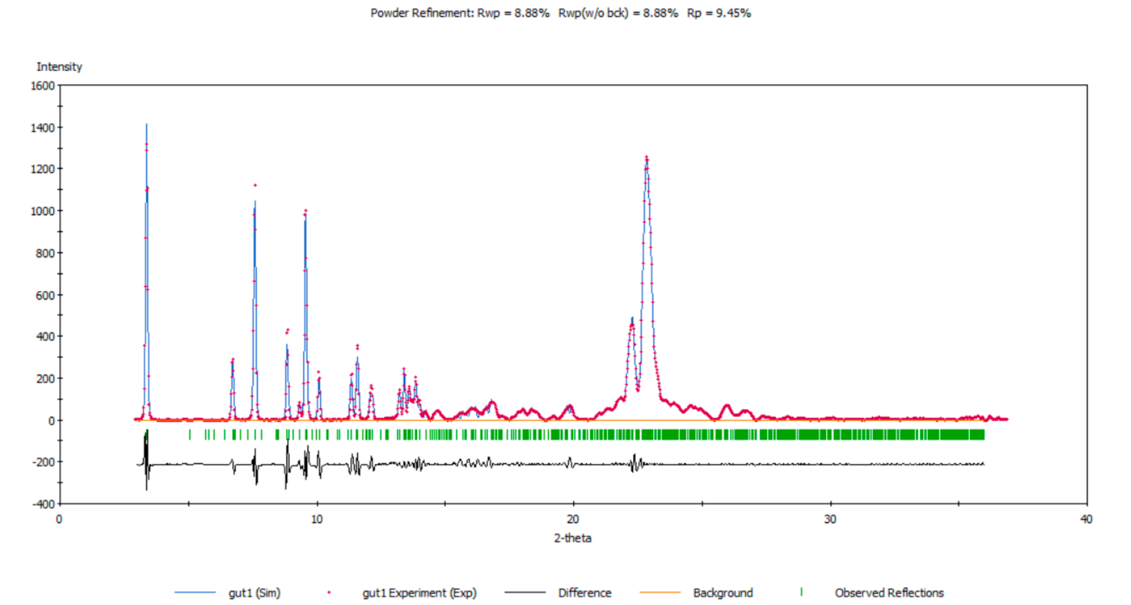
</td></tr>
</table>

Bu16	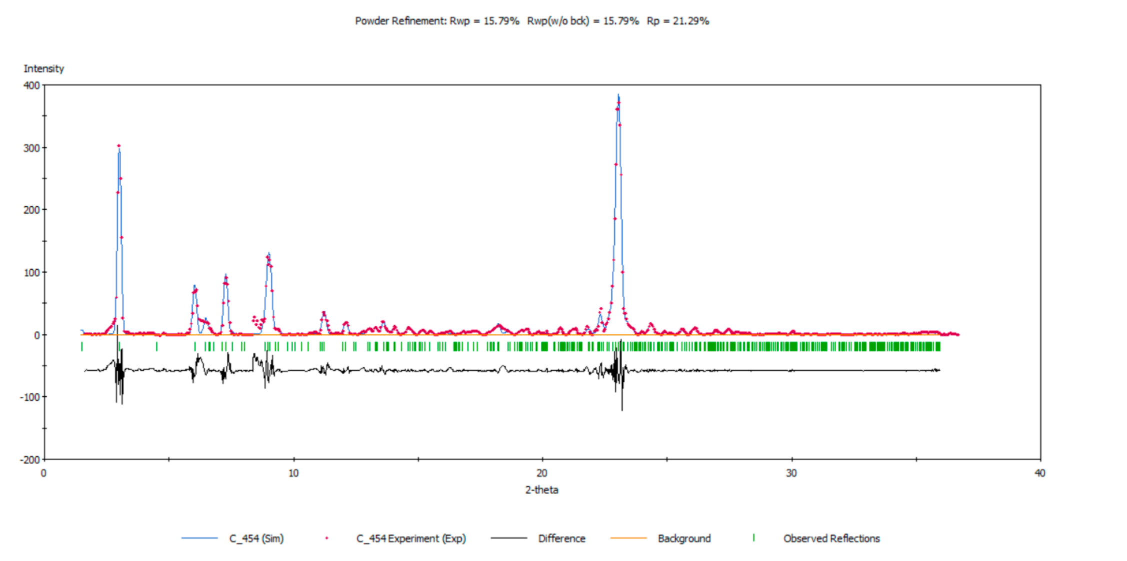
Bu18	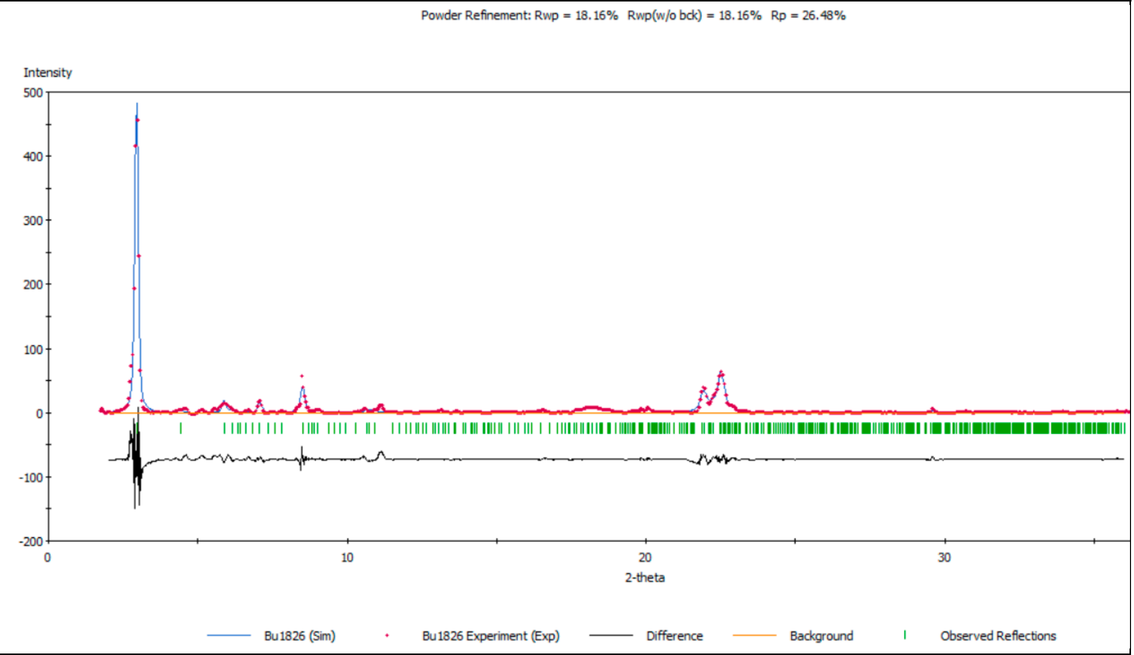

Pawley refinement data of compound **Bu6**

Bu6	h	k	l	dhkl	2-theta	I / I max	Multiplic-ity
	2	0	0	18.6	5.0	56.0	2
	0	0	1	13.0	7.1	100.0	2
	1	0	-1	12.7	7.3	32.9	2
	3	0	0	12.4	7.5	1.5	2
	2	0	-1	11.3	8.3	2.7	2
	1	1	-1	10.2	9.1	4.7	4
	2	0	1	10.1	9.2	4.4	2
	3	1	0	10.0	9.3	7.5	4
	4	0	0	9.3	10.1	34.6	2
	3	0	1	8.5	11.0	6.6	2
	1	2	0	8.4	11.2	2.5	4
	4	1	0	8.2	11.5	3.7	4

https://doi.org/10.3390/molecules31030401

	2	2	0	7.8	12.0	13.4	4
	4	0	1	7.2	13.1	9.7	2
	1	2	-1	7.1	13.2	1.1	4
	2	2	-1	6.8	13.7	6.1	4
	4	1	1	6.6	14.2	2.3	4
	1	0	2	6.3	15.0	6.5	2
	1	1	-2	6.1	15.4	1.8	4
	0	3	0	5.7	16.4	7.6	2
	3	1	-2	5.7	16.5	4.8	4
	1	3	0	5.7	16.6	1.6	4
	6	0	1	5.4	17.6	2.3	2
	3	1	2	5.2	18.0	2.1	4
	1	3	-1	5.2	18.0	24.4	4
	0	2	2	5.2	18.2	1.4	4
	1	3	1	5.2	18.3	3.6	4
	6	1	1	5.1	18.4	8.1	4
	2	3	-1	5.1	18.5	1.5	4
	6	2	0	5.0	18.8	1.3	4
	3	2	-2	4.9	19.1	1.3	4
	6	2	-1	4.8	19.5	6.1	4
	4	2	-2	4.7	20.0	1.4	4
	4	3	-1	4.7	20.3	4.0	4
	8	0	-1	4.5	20.8	5.8	2
	8	1	0	4.5	21.1	2.2	4
	8	1	-1	4.4	21.6	6.7	4
	1	3	-2	4.3	22.0	6.3	4
	2	3	-2	4.3	22.2	6.5	4
	6	0	2	4.3	22.3	1.0	2
	1	0	3	4.3	22.3	2.7	2
	3	0	-3	4.2	22.3	6.8	2
	1	3	2	4.2	22.3	5.7	4
	7	1	-2	4.2	22.4	7.9	4
	6	3	0	4.2	22.5	5.6	4
	3	3	-2	4.2	22.8	67.2	4
	6	1	2	4.1	22.9	16.2	4
	4	0	-3	4.1	23.0	24.4	2
	8	1	1	4.1	23.1	17.7	4
	6	3	-1	4.1	23.1	11.0	4
	0	4	1	4.1	23.2	24.4	4
	1	4	-1	4.1	23.2	9.2	4
	3	4	0	4.1	23.3	5.1	4
	1	4	1	4.0	23.4	4.4	4
	4	3	-2	4.0	23.6	5.3	4
	2	4	-1	4.0	23.6	4.8	4

	2	1	3	4.0	23.6	8.0	4
	7	0	2	3.9	24.3	4.4	2
	7	3	0	3.9	24.3	7.8	4
	6	0	-3	3.8	25.3	1.2	2
	4	4	1	3.7	25.7	1.2	4
	10	0	-1	3.7	25.8	1.2	2
	9	2	-1	3.7	25.8	1.3	4
	6	1	-3	3.7	25.9	4.3	4
	6	3	-2	3.7	25.9	3.3	4
	8	3	0	3.6	26.3	5.9	4
	10	1	-1	3.6	26.4	1.4	4
	3	2	3	3.6	26.4	1.1	4
	8	0	2	3.6	26.4	1.2	2
	0	4	2	3.6	26.5	1.8	4
	4	2	3	3.5	27.5	1.0	4
	6	2	-3	3.4	27.6	1.9	4
	0	5	0	3.4	27.6	1.7	2
	1	5	0	3.4	27.7	1.9	4
	4	4	-2	3.4	27.8	1.9	4
	4	3	-3	3.3	28.5	1.7	4
	9	0	-3	3.2	30.0	1.1	2
	5	0	-4	3.1	30.6	1.7	2

Pawley refinement data of compound **Bu8**

Bu8	h	k	l	dhkl	2-theta	I / I max	Multiplic-ity
	2	0	0	20.3	4.6	24.5	2
	2	1	0	11.9	7.9	16.3	4
	2	0	1	11.8	8.0	100.0	2
	0	1	1	10.5	9.0	1.1	4
	3	0	-1	10.3	9.2	4.5	2
	1	1	1	10.1	9.3	2.3	4
	3	1	0	10.0	9.4	2.2	4
	2	1	-1	9.5	10.0	3.5	4
	2	1	1	9.2	10.2	12.6	4
	3	1	-1	8.4	11.2	19.9	4
	4	1	0	8.4	11.3	14.4	4
	4	0	1	8.3	11.4	8.9	2
	3	1	1	8.2	11.5	2.9	4
	5	0	0	8.1	11.6	6.1	2
	0	0	2	7.5	12.6	3.5	2
	1	0	-2	7.4	12.7	3.0	2
	0	2	0	7.4	12.8	2.8	2
	1	0	2	7.3	12.9	28.9	2

https://doi.org/10.3390/molecules31030401

	5	0	-1	7.3	12.9	10.4	2
	1	2	0	7.3	13.0	13.8	4
	4	1	1	7.2	13.1	8.7	4
	5	1	0	7.1	13.2	6.7	4
	2	2	0	6.9	13.6	5.1	4
	6	0	0	6.8	13.9	5.0	2
	3	0	-2	6.7	14.1	14.3	2
	1	1	-2	6.6	14.2	3.2	4
	3	2	0	6.5	14.6	1.3	4
	2	2	-1	6.3	14.9	16.4	4
	6	0	-1	6.3	15.0	9.2	2
	4	2	-1	5.6	16.9	13.4	4
	5	2	0	5.5	17.3	3.7	4
	0	2	2	5.3	18.0	3.6	4
	1	2	-2	5.2	18.0	11.7	4
	1	2	2	5.2	18.2	5.9	4
	7	1	-1	5.2	18.3	2.1	4
	2	2	-2	5.1	18.4	2.6	4
	5	2	1	5.1	18.6	1.6	4
	5	1	2	5.1	18.6	2.6	4
	2	2	2	5.1	18.7	3.0	4
	3	2	-2	5.0	19.1	25.5	4
	1	0	3	4.9	19.1	12.9	2
	6	0	2	4.9	19.2	1.8	2
	1	1	-3	4.7	20.0	1.4	4
	7	0	-2	4.7	20.1	1.9	2
	0	3	1	4.7	20.2	3.8	4
	2	1	-3	4.7	20.3	10.9	4
	7	2	0	4.6	20.7	1.8	4
	2	3	1	4.5	20.8	7.6	4
	9	0	0	4.5	20.9	1.5	2
	7	1	-2	4.5	21.2	5.6	4
	3	3	-1	4.4	21.3	7.2	4
	4	0	3	4.4	21.4	5.5	2
	9	0	1	4.3	22.1	8.9	2
	4	3	-1	4.3	22.2	13.7	4
	4	1	3	4.2	22.4	31.4	4
	4	3	1	4.2	22.4	33.2	4
	6	2	-2	4.2	22.4	80.0	4
	5	3	0	4.2	22.5	16.3	4
	9	1	-1	4.2	22.5	48.5	4
	8	2	0	4.2	22.6	2.1	4
	0	2	3	4.1	22.9	1.3	4
	1	2	-3	4.1	22.9	1.4	4

	1	2	3	4.1	23.1	5.7	4
	1	3	-2	4.1	23.1	34.8	4
	6	2	2	4.1	23.1	10.4	4
	2	2	-3	4.1	23.2	2.8	4
	10	0	0	4.1	23.3	6.1	2
	2	3	-2	4.0	23.4	14.8	4
	5	1	3	4.0	23.5	2.7	4
	2	3	2	4.0	23.6	7.1	4
	3	2	-3	4.0	23.7	7.5	4
	8	1	2	4.0	23.8	1.4	4
	10	0	-1	4.0	23.9	2.2	2
	6	1	-3	4.0	23.9	2.6	4
	9	0	-2	3.9	24.0	1.4	2
	3	3	2	3.9	24.2	1.8	4
	7	0	-3	3.9	24.5	46.3	2
	9	1	-2	3.8	24.9	1.3	4
	6	1	3	3.8	24.9	1.6	4
	5	2	-3	3.7	25.3	1.5	4
	8	2	-2	3.7	25.6	2.6	4
	5	3	-2	3.7	25.6	13.9	4
	0	4	0	3.7	25.7	3.7	2
	9	1	2	3.7	25.8	1.4	4
	1	1	-4	3.6	26.1	6.0	4
	2	4	0	3.6	26.2	7.7	4
	7	3	1	3.6	26.2	4.6	4
	7	1	3	3.6	26.4	6.8	4
	6	2	-3	3.6	26.4	14.6	4
	8	1	-3	3.5	26.8	2.0	4
	8	0	3	3.5	27.2	1.5	2
	1	3	3	3.5	27.3	3.5	4
	9	2	2	3.4	28.1	6.0	4
	11	0	-2	3.4	28.1	1.1	2
	4	4	1	3.4	28.2	1.8	4
	5	4	0	3.4	28.3	1.4	4
	0	4	2	3.3	28.7	1.9	4
	6	4	-1	3.2	29.9	1.6	4
	10	2	2	3.2	30.0	4.2	4
	8	0	-4	3.1	30.9	1.5	2
	12	3	0	2.8	34.2	2.0	4
	12	0	3	2.8	34.7	1.3	2

Pawley refinement data of compound **Bu10**

Bu10	h	k	l	dhkl	2-theta	I / I max	Multiplicity

	2	0	0	23.3	4.0	100.0	2
	2	1	0	12.3	7.6	96.1	4
	3	0	-1	12.2	7.7	27.2	2
	0	1	1	11.0	8.6	30.7	4
	1	1	-1	10.9	8.6	13.0	4
	2	1	-1	10.3	9.2	3.4	4
	4	0	-1	10.2	9.2	1.4	2
	2	1	1	9.7	9.7	17.9	4
	4	0	1	9.1	10.4	1.9	2
	4	1	0	9.1	10.4	42.6	4
	0	0	2	8.4	11.1	8.4	2
	2	0	-2	8.3	11.4	27.8	2
	1	0	2	8.1	11.6	3.9	2
	3	0	-2	7.8	12.0	2.0	2
	6	0	0	7.8	12.1	15.6	2
	4	1	1	7.7	12.2	4.8	4
	2	0	2	7.6	12.3	13.5	2
	0	1	2	7.3	12.9	9.9	4
	4	0	-2	7.3	13.0	3.4	2
	0	2	0	7.2	13.0	9.3	2
	2	1	-2	7.2	13.1	4.7	4
	1	2	0	7.2	13.2	9.2	4
	2	2	0	6.9	13.6	5.7	4
	3	1	-2	6.9	13.7	3.1	4
	6	1	0	6.8	13.8	1.0	4
	2	1	2	6.8	13.9	4.9	4
	6	0	1	6.7	14.0	1.9	2
	5	0	-2	6.7	14.1	1.0	2
	7	0	0	6.7	14.2	1.1	2
	1	2	-1	6.6	14.2	1.3	4
	4	1	-2	6.5	14.5	12.0	4
	4	0	2	6.5	14.6	9.0	2
	7	0	-1	6.5	14.6	6.2	2
	2	2	1	6.3	14.9	2.9	4
	4	2	0	6.2	15.3	2.9	4
	6	1	1	6.1	15.4	4.9	4
	6	0	-2	6.1	15.5	2.4	2
	7	0	1	5.9	15.9	2.6	2
	4	1	2	5.9	16.0	10.3	4
	7	1	-1	5.9	16.0	4.2	4
	8	0	0	5.8	16.2	2.1	2
	1	0	-3	5.7	16.6	2.1	2
	4	2	1	5.7	16.7	3.4	4
	2	0	-3	5.6	16.8	1.2	2

https://doi.org/10.3390/molecules31030401

	1	2	2	5.4	17.4	5.5	4
	8	1	0	5.4	17.5	3.5	4
	0	1	3	5.2	18.0	8.8	4
	7	1	-2	5.2	18.2	1.1	4
	4	2	-2	5.1	18.4	1.1	4
	9	0	-1	5.1	18.4	1.0	2
	8	0	-2	5.1	18.6	3.3	2
	3	2	2	5.1	18.7	7.3	4
	4	1	-3	5.0	18.9	9.8	4
	8	1	1	5.0	18.9	5.0	4
	7	0	2	4.9	19.1	9.9	2
	6	2	-2	4.7	20.3	1.3	4
	10	0	-1	4.6	20.4	1.2	2
	7	2	1	4.6	20.6	2.1	4
	5	0	3	4.6	20.6	1.5	2
	2	3	1	4.5	20.9	7.2	4
	3	3	-1	4.5	21.0	6.7	4
	4	3	0	4.5	21.2	4.0	4
	0	2	3	4.4	21.3	7.2	4
	10	1	0	4.4	21.3	5.8	4
	10	1	-1	4.4	21.4	2.4	4
	8	0	-3	4.3	21.9	2.1	2
	6	0	3	4.3	21.9	2.8	2
	10	0	-2	4.3	22.0	10.6	2
	2	2	3	4.3	22.0	19.3	4
	8	2	1	4.3	22.1	11.4	4
	4	3	1	4.3	22.2	11.2	4
	1	0	-4	4.3	22.2	1.3	2
	2	0	-4	4.2	22.3	20.4	2
	11	0	0	4.2	22.4	29.6	2
	0	0	4	4.2	22.4	36.3	2
	5	3	-1	4.2	22.5	23.2	4
	1	3	-2	4.2	22.5	18.2	4
	0	3	2	4.2	22.6	13.4	4
	9	2	-1	4.2	22.6	35.3	4
	2	3	-2	4.2	22.7	12.3	4
	10	1	1	4.2	22.7	8.3	4
	3	2	3	4.2	22.7	62.0	4
	8	2	-2	4.2	22.7	5.3	4
	1	3	2	4.2	22.8	29.0	4
	8	1	-3	4.1	22.9	21.0	4
	10	1	-2	4.1	22.9	22.5	4
	3	3	-2	4.1	23.0	8.5	4
	6	3	0	4.1	23.1	7.5	4

https://doi.org/10.3390/molecules31030401

	2	3	2	4.1	23.2	25.3	4	
	2	1	-4	4.1	23.2	7.0	4	
	2	0	4	4.1	23.3	27.9	2	
	0	1	4	4.1	23.3	11.7	4	
	3	1	-4	4.0	23.4	6.6	4	
	4	3	-2	4.0	23.5	14.4	4	
	1	1	4	4.0	23.7	1.9	4	
	3	0	4	4.0	23.9	1.4	2	
	6	3	1	3.9	24.1	7.3	4	
	2	1	4	3.9	24.2	2.7	4	
	10	2	-1	3.9	24.3	10.2	4	
	5	1	-4	3.9	24.4	4.6	4	
	11	1	-2	3.8	24.7	1.3	4	
	4	0	4	3.8	24.8	1.9	2	
	12	1	-1	3.8	25.3	4.9	4	
	5	3	2	3.7	25.3	1.9	4	
	10	2	1	3.7	25.4	4.4	4	
	8	3	0	3.7	25.5	7.2	4	
	8	2	-3	3.7	25.6	4.5	4	
	6	2	3	3.7	25.6	5.7	4	
	10	2	-2	3.7	25.6	4.4	4	
	12	0	-2	3.7	25.6	3.3	2	
	10	1	-3	3.7	25.6	1.6	4	
	8	3	-1	3.7	25.7	4.4	4	
	1	3	-3	3.7	25.8	3.1	4	
	2	2	-4	3.7	25.9	3.8	4	
	1	2	4	3.6	26.3	1.2	4	
	6	3	2	3.6	26.3	3.2	4	
	4	2	-4	3.6	26.4	3.1	4	
	12	1	-2	3.6	26.5	3.6	4	
	8	3	1	3.6	26.6	1.7	4	
	2	2	4	3.5	26.7	3.3	4	
	9	2	-3	3.5	26.8	7.3	4	
	3	4	0	3.5	26.9	1.3	4	
	4	4	0	3.5	27.4	2.0	4	
	13	0	-2	3.5	27.5	1.5	2	
	6	2	-4	3.4	27.6	2.0	4	
	6	1	4	3.4	27.7	2.6	4	
	12	2	-1	3.4	27.7	1.0	4	
	4	4	1	3.4	28.2	1.3	4	
	10	3	-1	3.3	28.4	1.8	4	
	1	4	-2	3.3	28.5	1.1	4	
	2	4	-2	3.3	28.6	1.3	4	
	4	1	-5	3.3	29.1	1.3	4	

https://doi.org/10.3390/molecules31030401

	h	k	l	dhkl	2-theta	I / I max	Multiplicity
	11	3	1	3.1	30.9	1.1	4
	15	0	1	3.0	31.9	1.1	2
	12	1	-4	3.0	31.9	1.6	4

Pawley refinement data of compound **Bu11**

Bu11	h	k	l	dhkl	2-theta	I / I max	Multiplic-ity
	2	0	0	23.9	2.4	24.5	2
	2	0	1	13.0	4.5	2.0	2
	2	1	0	12.7	4.6	36.2	4
	3	0	-1	12.5	4.7	2.9	2
	4	0	0	12.0	4.9	3.7	2
	0	1	1	11.2	5.2	24.9	4
	2	1	-1	10.5	5.5	2.1	4
	4	0	-1	10.4	5.6	1.3	2
	2	1	1	9.8	5.9	4.4	4
	4	1	0	9.3	6.2	16.0	4
	1	0	-2	8.5	6.8	1.4	2
	0	0	2	8.5	6.9	4.4	2
	2	0	-2	8.3	7.0	23.8	2
	5	1	0	8.1	7.2	3.6	4
	6	0	0	8.0	7.3	4.8	2
	4	1	1	7.8	7.4	1.7	4
	2	0	2	7.7	7.6	9.2	2
	0	1	2	7.4	7.9	7.2	4
	2	1	-2	7.3	8.0	7.4	4
	3	0	2	7.1	8.2	1.7	2
	0	2	1	6.8	8.5	1.7	4
	2	1	2	6.8	8.5	1.8	4
	1	2	-1	6.8	8.6	1.2	4
	4	1	-2	6.6	8.8	11.7	4
	6	1	1	6.2	9.3	4.6	4
	8	0	0	6.0	9.7	8.5	2
	8	1	-1	5.5	10.6	5.3	4
	6	0	2	5.4	10.7	8.0	2
	2	1	-3	5.3	11.0	4.2	4
	9	0	-1	5.3	11.0	1.7	2
	0	1	3	5.3	11.0	1.4	4
	8	0	-2	5.2	11.2	1.6	2
	3	2	2	5.1	11.3	3.6	4
	6	1	2	5.1	11.4	3.0	4
	6	2	1	5.1	11.5	2.1	4
	7	2	0	5.0	11.6	2.9	4
	5	2	-2	5.0	11.6	9.5	4

	2	1	3	5.0	11.6	4.2	4
	6	1	-3	4.7	12.5	3.5	4
	8	2	-1	4.6	12.6	4.0	4
	3	3	-1	4.6	12.6	2.3	4
	4	1	3	4.6	12.6	1.7	4
	8	0	2	4.6	12.7	1.1	2
	10	1	-1	4.5	12.8	1.5	4
	4	3	-1	4.5	13.0	7.2	4
	5	3	0	4.4	13.2	1.4	4
	8	2	1	4.4	13.3	4.9	4
	4	3	1	4.4	13.3	6.8	4
	6	0	3	4.3	13.4	1.9	2
	2	2	3	4.3	13.4	8.2	4
	5	3	-1	4.3	13.4	4.0	4
	10	1	1	4.3	13.7	12.0	4
	2	0	-4	4.3	13.7	15.1	2
	1	0	-4	4.3	13.7	13.3	2
	10	1	-2	4.2	13.7	100.0	4
	8	1	-3	4.2	13.8	4.5	4
	6	3	0	4.2	13.8	24.6	4
	3	2	3	4.2	13.8	4.2	4
	3	3	-2	4.2	13.8	1.0	4
	6	1	3	4.2	14.0	1.3	4
	6	3	-1	4.2	14.0	3.2	4
	4	0	-4	4.2	14.0	3.9	2
	1	0	4	4.2	14.0	5.0	2
	7	2	2	4.2	14.0	3.2	4
	9	0	-3	4.2	14.0	16.2	2
	4	3	-2	4.1	14.1	2.9	4
	6	2	-3	4.1	14.2	1.9	4
	2	1	-4	4.1	14.2	6.0	4
	1	1	-4	4.1	14.2	5.7	4
	11	0	-2	4.1	14.2	4.8	2
	9	1	2	4.1	14.3	1.2	4
	7	0	3	4.1	14.3	1.1	2
	9	2	1	4.1	14.3	1.8	4
	11	0	1	4.1	14.3	3.2	2
	5	0	-4	4.1	14.3	5.4	2
	3	3	2	4.1	14.3	4.1	4
	2	0	4	4.1	14.3	8.6	2
	4	2	3	4.1	14.3	1.1	4
	12	0	-1	4.0	14.6	1.6	2
	9	1	-3	4.0	14.6	1.9	4
	12	0	0	4.0	14.6	2.5	2

	6	0	-4	4.0	14.7	1.9	2
	3	0	4	4.0	14.7	1.2	2
	7	1	3	3.9	14.8	1.1	4
	11	1	1	3.9	14.8	4.3	4
	5	1	-4	3.9	14.8	1.7	4
	2	1	4	3.9	14.9	1.3	4
	12	1	-1	3.9	15.1	1.5	4
	7	3	1	3.8	15.2	2.4	4
	7	0	-4	3.8	15.2	3.0	2
	4	0	4	3.8	15.2	1.8	2
	6	1	-4	3.8	15.2	1.2	4
	10	1	-3	3.8	15.4	1.9	4
	12	0	1	3.8	15.5	6.4	2
	1	4	0	3.7	15.7	19.5	4
	7	1	-4	3.7	15.7	3.0	4
	11	0	2	3.7	15.9	2.3	2
	6	3	2	3.7	15.9	2.4	4
	13	0	-2	3.6	16.4	12.4	2
	4	4	-1	3.5	16.6	1.3	4
	8	2	3	3.4	17.0	1.5	4

Pawley refinement data of compound **Bu12**

Bu12	h	k	l	dhkl	2-theta	I / I max	Multiplicity
	2	0	0	25.61	3.72	92.55	2
	4	0	0	12.80	7.40	19.98	2
	2	1	0	12.33	7.69	100.00	4
	0	1	1	10.75	8.81	29.53	4
	2	1	-1	10.13	9.34	4.75	4
	2	1	1	9.71	9.75	15.07	4
	4	1	0	9.47	10.00	60.35	4
	6	0	0	8.54	11.09	11.62	2
	4	1	-1	8.49	11.15	8.43	4
	0	0	2	8.34	11.35	5.87	2
	5	1	0	8.28	11.43	2.74	4
	2	0	-2	8.15	11.60	19.27	2
	1	0	2	8.11	11.66	5.23	2
	4	1	1	8.00	11.83	4.82	4
	2	0	2	7.72	12.26	5.57	2
	5	1	-1	7.65	12.37	2.31	4
	6	1	0	7.30	12.97	4.91	4
	0	1	2	7.17	13.19	10.53	4
	2	1	-2	7.05	13.41	12.01	4
	0	2	0	7.03	13.46	2.89	2

	3	1	-2	6.81	13.90	2.13	4
	5	0	-2	6.78	13.95	6.62	2
	2	1	2	6.77	13.98	4.81	4
	4	0	2	6.71	14.10	3.99	2
	3	2	0	6.50	14.55	11.50	4
	1	2	-1	6.46	14.65	1.75	4
	4	2	-1	5.87	16.13	1.18	4
	8	1	0	5.83	16.24	3.39	4
	5	2	0	5.80	16.33	1.52	4
	7	0	-2	5.77	16.40	2.07	2
	6	2	0	5.43	17.45	2.07	4
	5	2	1	5.39	17.57	2.95	4
	1	1	-3	5.19	18.25	2.95	4
	0	1	3	5.17	18.32	1.18	4
	2	1	-3	5.15	18.37	2.41	4
	10	0	0	5.12	18.49	2.12	2
	6	2	1	5.07	18.67	1.03	4
	4	2	-2	5.07	18.70	1.30	4
	3	1	3	4.83	19.60	1.74	4
	10	1	0	4.81	19.69	1.62	4
	7	2	1	4.77	19.88	2.61	4
	10	1	-1	4.73	20.01	2.46	4
	8	2	0	4.73	20.02	2.19	4
	6	2	-2	4.68	20.26	2.57	4
	8	2	-1	4.64	20.43	1.78	4
	8	1	2	4.60	20.62	3.95	4
	10	0	-2	4.56	20.80	12.69	2
	3	3	0	4.52	20.97	7.67	4
	1	3	1	4.49	21.13	1.98	4
	7	2	-2	4.46	21.25	2.39	4
	6	2	2	4.43	21.40	2.76	4
	7	1	-3	4.40	21.52	1.59	4
	1	2	-3	4.37	21.69	1.13	4
	2	2	-3	4.35	21.79	4.60	4
	3	3	1	4.34	21.87	1.98	4
	10	1	-2	4.33	21.88	3.62	4
	1	2	3	4.32	21.96	3.90	4
	4	3	-1	4.29	22.11	26.10	4
	12	0	0	4.27	22.22	16.12	2
	2	2	3	4.25	22.33	6.13	4
	8	2	-2	4.24	22.35	4.86	4
	4	2	-3	4.22	22.46	1.70	4
	9	2	1	4.20	22.58	8.25	4
	10	0	2	4.19	22.62	14.86	2

	11	1	1	4.18	22.68	27.57	4
	5	3	-1	4.17	22.76	48.59	4
	3	2	3	4.15	22.84	22.64	4
	3	0	-4	4.14	22.92	4.87	2
	1	0	4	4.12	23.00	7.93	2
	6	3	0	4.11	23.09	7.00	4
	5	3	1	4.09	23.19	5.23	4
	4	0	-4	4.08	23.27	3.88	2
	12	1	-1	4.05	23.43	2.94	4
	4	2	3	4.04	23.50	3.35	4
	6	3	-1	4.03	23.54	2.37	4
	9	2	-2	4.03	23.55	3.35	4
	10	1	2	4.02	23.62	5.16	4
	2	3	2	4.01	23.69	2.58	4
	9	1	-3	4.00	23.72	1.98	4
	5	0	-4	3.99	23.77	3.02	2
	1	1	4	3.96	23.98	1.26	4
	3	3	2	3.93	24.13	2.43	4
	4	1	-4	3.92	24.24	1.82	4
	2	1	4	3.90	24.36	1.97	4
	11	2	0	3.88	24.46	2.82	4
	7	2	-3	3.87	24.52	2.42	4
	5	3	-2	3.86	24.62	1.60	4
	3	1	4	3.82	24.88	1.64	4
	10	1	-3	3.81	24.95	1.35	4
	13	1	0	3.79	25.04	1.98	4
	8	2	-3	3.73	25.45	2.86	4
	4	1	4	3.72	25.52	4.36	4
	11	2	1	3.72	25.55	2.80	4
	13	0	-2	3.70	25.70	5.47	2
	9	1	3	3.67	25.88	2.83	4
	10	2	2	3.60	26.39	1.34	4
	0	2	4	3.59	26.51	1.74	4
	2	3	-3	3.58	26.57	1.75	4
	3	2	-4	3.57	26.65	2.84	4
	1	2	4	3.56	26.72	1.57	4
	3	3	-3	3.55	26.78	1.28	4
	5	2	-4	3.47	27.39	1.48	4
	1	4	-1	3.44	27.68	1.58	4
	6	4	0	3.25	29.29	1.07	4

Pawley refinement data of compound **Bu14**

Bu14	h	k	l	dhkl	2-theta	I / I max	Multiplicity

	2	0	0	28.0	3.4	100.0	2
	4	0	0	14.0	6.7	19.8	2
	2	1	0	12.4	7.6	74.5	4
	0	1	1	10.7	8.8	25.7	4
	1	1	-1	10.6	8.9	2.0	4
	2	1	-1	10.1	9.3	4.8	4
	4	1	0	9.9	9.6	65.3	4
	2	1	1	9.8	9.6	9.1	4
	6	0	0	9.3	10.1	14.0	2
	4	1	1	8.3	11.3	15.0	4
	1	0	2	8.2	11.6	10.8	2
	2	0	-2	8.1	11.6	14.1	2
	3	0	-2	7.8	12.1	8.1	2
	6	1	0	7.7	12.2	7.5	4
	0	1	2	7.1	13.2	10.1	4
	7	0	1	7.0	13.4	18.0	2
	7	1	0	6.9	13.6	11.4	4
	6	1	1	6.9	13.7	4.8	4
	2	1	2	6.8	13.8	7.4	4
	3	1	-2	6.8	13.9	8.8	4
	2	2	0	6.7	14.0	5.5	4
	8	0	-1	6.6	14.2	3.6	2
	5	0	2	6.5	14.6	1.9	2
	6	0	-2	6.4	14.7	2.7	2
	0	2	1	6.4	14.7	1.8	4
	1	2	-1	6.4	14.8	1.4	4
	3	2	-1	6.1	15.5	2.0	4
	6	0	2	6.0	15.7	3.2	2
	9	0	-1	6.0	15.8	2.8	2
	5	2	0	5.9	16.0	4.8	4
	5	1	2	5.9	16.1	1.6	4
	6	1	-2	5.8	16.2	4.6	4
	4	2	1	5.8	16.4	2.7	4
	9	0	1	5.7	16.5	2.1	2
	9	1	0	5.7	16.6	4.8	4
	5	2	-1	5.6	16.8	7.8	4
	7	0	2	5.6	16.9	4.5	2
	6	2	0	5.6	17.0	2.7	4
	9	1	-1	5.5	17.2	1.6	4
	1	2	-2	5.3	17.7	1.6	4
	4	0	-3	5.3	17.9	3.1	2
	7	2	0	5.2	18.0	4.2	4
	6	2	1	5.2	18.1	1.3	4
	7	1	2	5.2	18.2	1.4	4

https://doi.org/10.3390/molecules31030401

	8	1	-2	5.2	18.3	3.7	4
	2	1	-3	5.1	18.4	2.7	4
	11	0	0	5.1	18.5	3.1	2
	3	2	2	5.1	18.6	1.6	4
	4	2	2	4.9	19.2	1.3	4
	9	0	2	4.8	19.6	2.1	2
	10	0	-2	4.8	19.7	1.9	2
	11	1	0	4.8	19.8	5.6	4
	4	1	3	4.7	19.9	6.3	4
	7	2	-2	4.5	20.9	1.0	4
	10	0	2	4.5	21.0	1.6	2
	11	0	-2	4.5	21.1	1.7	2
	7	1	-3	4.5	21.2	1.6	4
	1	3	-1	4.4	21.3	1.9	4
	1	3	1	4.4	21.3	2.1	4
	2	3	-1	4.4	21.4	2.8	4
	2	3	1	4.4	21.6	3.8	4
	10	2	0	4.4	21.7	7.0	4
	1	2	-3	4.3	21.8	4.9	4
	0	2	3	4.3	21.8	2.4	4
	2	2	-3	4.3	21.9	5.2	4
	10	1	2	4.3	22.1	6.1	4
	8	1	-3	4.3	22.1	8.3	4
	4	3	-1	4.3	22.2	4.3	4
	11	1	-2	4.3	22.2	24.5	4
	13	0	-1	4.2	22.3	42.1	2
	4	3	1	4.2	22.4	14.2	4
	12	0	-2	4.2	22.6	6.8	2
	3	2	3	4.2	22.7	42.3	4
	8	2	2	4.2	22.8	64.5	4
	6	3	0	4.1	22.8	67.9	4
	1	0	4	4.1	22.9	50.2	2
	3	0	-4	4.1	23.0	13.0	2
	13	1	0	4.1	23.0	25.0	4
	5	3	1	4.1	23.0	10.7	4
	13	0	1	4.1	23.0	22.2	2
	9	1	-3	4.1	23.1	8.6	4
	10	0	-3	4.1	23.2	12.4	2
	4	2	3	4.1	23.2	1.5	4
	2	0	4	4.1	23.2	3.0	2
	4	0	-4	4.1	23.3	9.1	2
	13	1	-1	4.1	23.3	2.7	4
	6	3	-1	4.0	23.4	3.4	4
	8	1	3	4.0	23.5	4.3	4

https://doi.org/10.3390/molecules31030401

	1	3	2	4.0	23.6	2.0	4
	12	1	-2	4.0	23.6	1.2	4
	6	2	-3	4.0	23.6	1.7	4
	3	0	4	4.0	23.6	1.3	2
	7	3	0	4.0	23.7	2.5	4
	6	3	1	4.0	23.7	1.5	4
	3	3	-2	4.0	23.8	2.6	4
	5	2	3	4.0	23.8	1.1	4
	9	2	2	4.0	23.9	2.2	4
	1	1	4	4.0	23.9	1.1	4
	14	0	-1	4.0	24.0	2.5	2
	11	2	1	3.9	24.1	3.6	4
	3	3	2	3.9	24.1	2.1	4
	2	1	4	3.9	24.2	1.8	4
	11	0	-3	3.9	24.4	2.8	2
	12	2	0	3.9	24.5	2.4	4
	7	3	1	3.9	24.5	3.6	4
	6	2	3	3.9	24.6	1.5	4
	4	3	2	3.8	24.6	1.8	4
	14	0	1	3.8	24.7	4.2	2
	12	2	-1	3.8	24.8	3.2	4
	7	0	-4	3.8	24.9	2.9	2
	8	3	-1	3.8	25.0	2.4	4
	4	1	4	3.8	25.1	1.1	4
	10	1	3	3.7	25.8	1.8	4
	6	3	2	3.7	25.9	2.2	4
	7	1	-4	3.7	25.9	1.6	4
	13	2	0	3.7	25.9	1.7	4
	9	2	-3	3.6	26.0	5.6	4
	11	0	3	3.6	26.1	3.9	2
	14	1	-2	3.6	26.5	1.5	4
	2	2	-4	3.6	26.6	2.4	4
	1	2	4	3.5	26.7	3.7	4
	13	2	1	3.5	26.9	3.1	4
	9	2	3	3.5	27.3	1.5	4
	9	3	-2	3.4	27.6	1.1	4
	10	1	-4	3.4	28.3	1.5	4
	7	3	-3	3.3	28.8	1.1	4
	1	1	6	2.7	35.3	1.3	4
	9	4	-3	2.7	35.4	1.1	4
	12	4	-2	2.7	35.8	1.1	4
	19	1	2	2.7	35.8	1.1	4

Pawley refinement data of compound **Bu16**

Bu16	h	k	l	dhkl	2-theta	I / I max	Multiplic-ity
	1	0	0	62.4	1.5	2.1	2
	2	0	0	31.2	3.0	93.1	2
	4	0	0	15.6	6.0	26.6	2
	0	1	0	14.6	6.5	8.5	2
	1	1	0	14.2	6.6	2.7	4
	2	0	1	13.0	7.3	33.4	4
	4	1	0	10.6	8.9	6.6	4
	4	0	1	10.5	9.0	24.1	4
	6	0	0	10.4	9.1	30.4	2
	0	1	1	10.2	9.3	1.7	4
	1	1	1	10.0	9.4	1.8	8
	6	0	1	8.4	11.2	12.8	4
	8	0	0	7.8	12.1	7.6	2
	0	2	0	7.3	13.0	2.0	2
	1	2	0	7.2	13.1	2.0	4
	0	0	2	7.1	13.3	2.3	2
	1	0	2	7.1	13.4	1.6	4
	2	0	2	6.9	13.6	7.1	4
	3	0	2	6.7	14.0	1.9	4
	7	1	1	6.7	14.1	2.7	8
	0	2	1	6.5	14.6	1.9	4
	1	2	1	6.4	14.7	2.4	8
	9	0	1	6.2	15.2	2.5	4
	3	1	2	6.1	15.5	1.6	8
	6	0	2	5.9	16.1	1.7	4
	7	2	0	5.6	16.8	1.8	4
	7	0	2	5.6	17.0	1.3	4
	6	2	1	5.5	17.2	1.8	8
	6	1	2	5.4	17.4	1.9	8
	8	0	2	5.3	18.0	1.5	4
	7	1	2	5.2	18.2	6.0	8
	0	2	2	5.1	18.6	2.9	4
	12	1	0	4.9	19.3	1.9	4
	12	0	1	4.9	19.4	1.1	4
	2	3	0	4.8	19.8	1.2	4
	1	3	1	4.6	20.7	2.9	8
	3	3	1	4.5	21.1	1.3	8
	14	0	0	4.5	21.3	2.7	2
	13	1	1	4.3	21.8	3.0	8
	14	0	1	4.3	22.3	6.6	4
	11	1	2	4.2	22.4	4.9	8
	7	0	3	4.2	22.7	5.1	4

	15	0	0	4.2	22.8	13.7	2
	6	1	3	4.1	22.9	42.0	8
	8	3	0	4.1	23.0	100.0	4
	9	2	2	4.1	23.1	43.8	8
	12	2	1	4.1	23.4	4.4	8
	7	1	3	4.0	23.6	2.4	8
	8	1	3	3.9	24.3	3.4	8
	16	0	0	3.9	24.3	2.0	2
	4	3	2	3.9	24.5	1.9	8
	5	3	2	3.8	24.9	1.5	8
	6	2	3	3.7	25.6	3.1	8
	16	1	1	3.6	26.1	2.1	8
	11	1	3	3.5	26.9	1.9	8
	18	0	0	3.5	27.4	1.8	2
	0	1	4	3.5	27.5	1.1	4
	12	3	2	3.2	30.0	1.6	8

Pawley refinement data of compound **Bu18**

Bu18	h	k	l	dhkl	2-theta	I / I max	Multiplic-ity
	2	0	0	31.9	2.9	100.0	2
	3	0	0	21.3	4.4	1.5	2
	4	0	0	15.9	5.9	4.6	2
	0	1	0	15.2	6.2	1.8	2
	2	0	1	13.3	7.1	4.6	4
	4	1	0	11.0	8.5	11.7	4
	1	1	1	10.4	9.0	1.2	8
	4	1	1	8.8	10.7	1.4	8
	6	0	1	8.6	10.9	1.8	4
	7	0	2	5.7	16.5	1.5	4
	12	0	0	5.3	17.7	0.9	2
	2	2	2	5.2	18.1	2.2	8
	8	2	1	5.2	18.2	1.5	8
	3	2	2	5.1	18.4	1.5	8
	3	0	3	4.7	19.8	1.2	4
	3	3	1	4.7	20.1	1.8	8
	7	0	3	4.3	21.9	13.7	4
	9	2	2	4.2	22.2	3.4	8
	14	1	1	4.2	22.5	4.9	8
	12	2	1	4.2	22.5	20.7	8
	0	3	2	4.2	22.6	1.6	4
	10	4	1	3.2	29.6	1.6	8

Figure S3. Pristine phase of compound Bu18

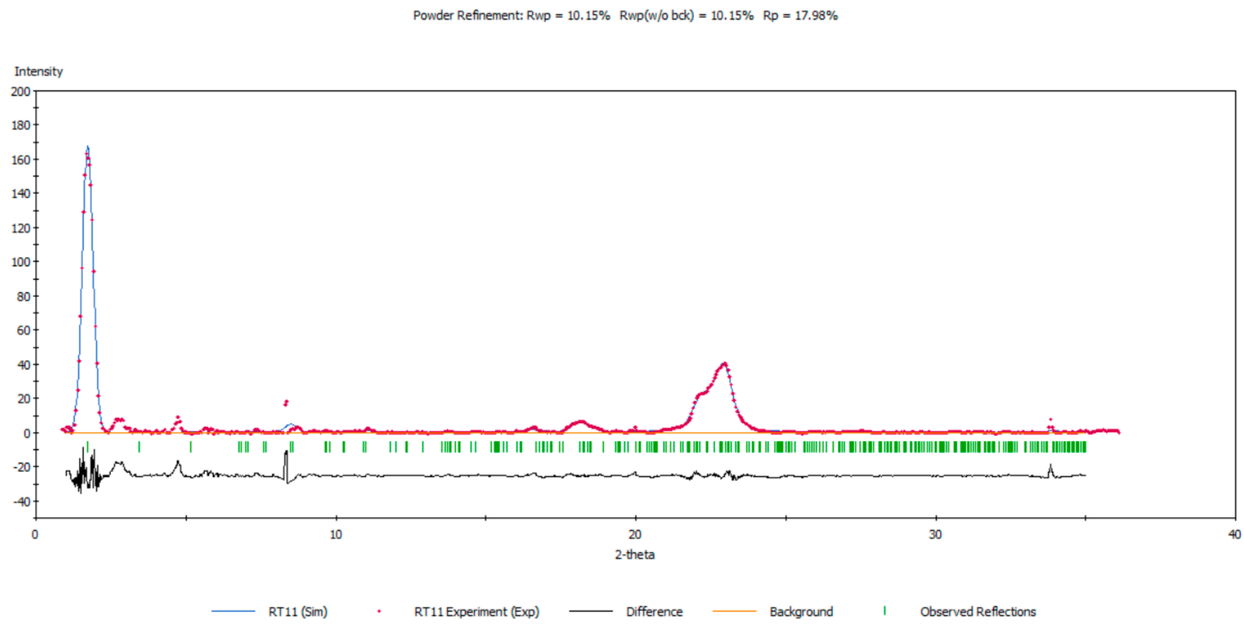

Compound Bu18 pristine phase was indexed as an orthorhombic P222 with parameters a=54.4 Å, b=13.6 Å c=13.8 Å.

Figure S4. WAXS for Bu11

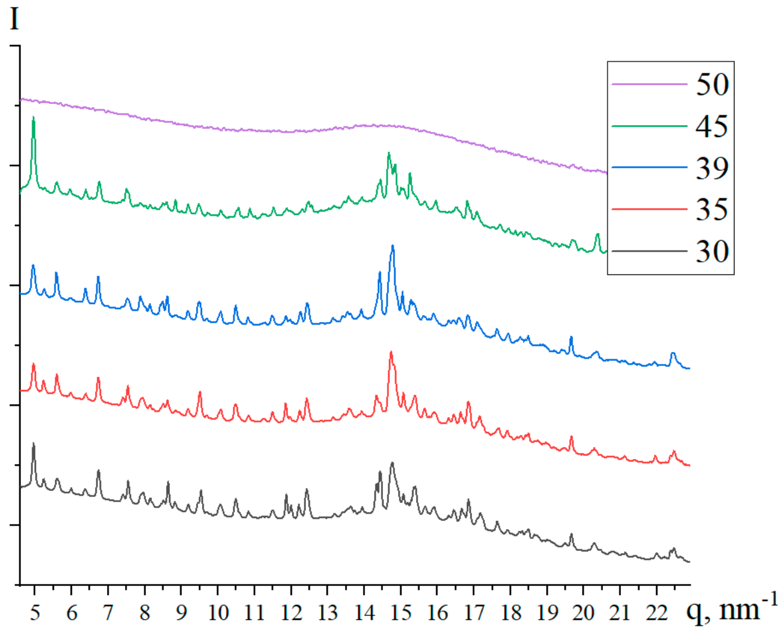

Figure S5. Comparison of molecular models of dipole(a), quadrupole(b) and octupole (c) of Bu11 and corresponding energy minimization plots.

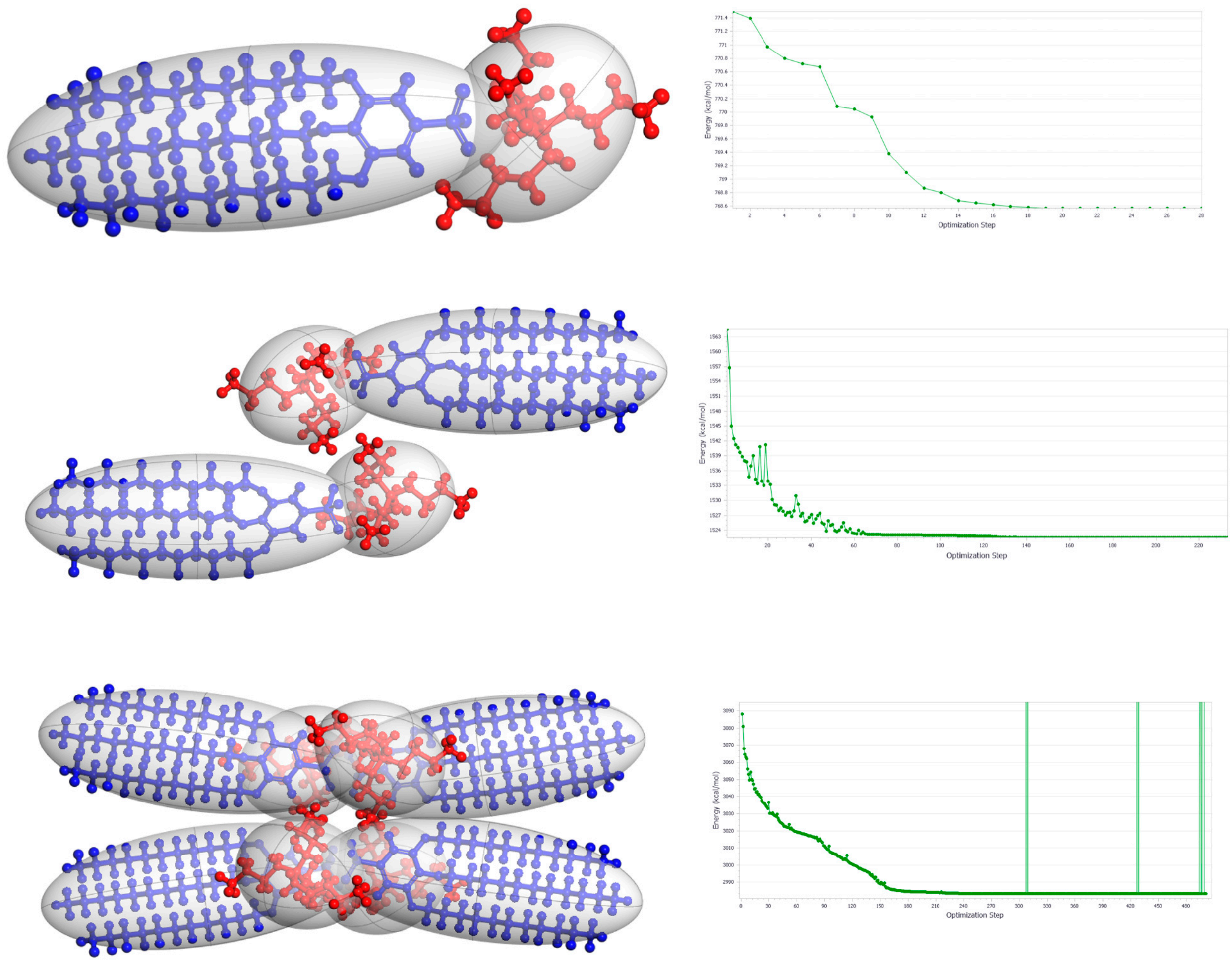

Energy estimation of dipole, quadrupole, octupole configurations was done using Forcite geometry optimization procedure with COMPASSIII (Version 1.2) forcefield[1,2] in the Biovia Materials Studio software package. While the motion groups of the tris(undecyloxy)benzenesulfonate and tetrabutylammonium were kept rigid, the charges were assigned by the forcefield. Smart convergence criteria were set as default: 2e-5 kcal/mol (energy), 0.001 kcal/mol/A (force), 1e-5 A (displacement). The total final energy conformations yielded 768 kcal/mol, 1522 kcal/mol and 2983 kcal/mol for dipole, quadrupole and octupole correspondingly. It was found that the total energy decreases per molecule (from 768 kcal/mol to 761 kcal/mol and to 745 kcal/mol). This occurs mainly due to electrostatic interactions: 4.7 kcal/mol for dipole, 0.4 kcal/mol for quadrupole and -6.3 kcal/mol for octupole.

1. Sun, H. COMPASS: An Ab Initio Force-Field Optimized for Condensed-Phase ApplicationsOverview with Details on Alkane and Benzene Compounds. *J. Phys. Chem. B* **1998**, *102*, 7338–7364, doi:10.1021/jp980939v.

2. Sun, H.; Ren, P.; Fried, J.R. The COMPASS Force Field: Parameterization and Validation for Phosphazenes. *Comput. Theor. Polym. Sci.* **1998**, *8*, 229–246, doi:10.1016/S1089-3156(98)00042-7.

EXPERIMENTAL SECTION

Materials

2-Ethylhexyl methacrylate (EHMA) (≥98%, Alfa Aesar), ethylene glycol dimethacrylate (EGDMA) (≥98%, Alfa Aesar), 2-hydroxyethyl methacrylate (HEMA) (≥97%, Alfa Aesar), n-hexyl methacrylate (HMA) (≥97%, Alfa Aesar), triethylene glycol dimethacrylate (TEGDMA) (99%, Sigma-Aldrich), benzene (≥99.7%, Sigma-Aldrich), N,N-dimethylformamide (DMF) (99.9%, Sigma-Aldrich), styrene (≥99.5%, Fluka), tetrahydrofuran (THF) (99.9%, Sigma-Aldrich), toluene (≥99.7 %, Fluka), acetone, methanol, n-hexane were used as received.

Synthesis

3,4,5-trihydroxybenzenesulfonic acid: Through 100mL of an aqueous solution of pyrogallol (10g, 0.08mol), Na_2SO_3 (25.2g, 0.2mol) and NaOH (3.3g, 0.08mol), air was strongly bubbled for 10h to form a dark solution. 50mL HCl (16%) were added and the mixture became continuously extracted with diethylether over 48h in a liquid-liquid extraction equipment. After addition of 51.2g (0.2mol) $Ba(OAc)_2$, dissolved in water, the precipitate was filtrated off and treated with sulfuric acid. The filtered solution was run subsequently over a pre-acidified ion-exchanger column (450g Amberlite IR 120 resin) to remove all metal ions. The strongly acidic solution was decolorized by boiling for 30min with 4g activated charcoal and the main amount of water was removed at reduced pressure on a rotary evaporator. After 3 times recrystallization from 100mL water at 0°C, the white crystals were dried over P_4O_{10} in vacuum for 48h to obtain a white powder.

Yield: 11.60g≅71% of theory ($C_6H_6O_6S$, 206.18 g/mol). M.p.: 39~40°C (by DSC). ^{1}H-NMR (D_2O, ppm): 6.911 (s, 2H, benzene-H); ^{13}C-NMR(D_2O, ppm): 106.19(2-benzene-C), 134.241(4-benzene–C), 135.742(1-benzene–C), 145.122(3- benzene –C).

Table S1. Synthetic conditions to prepare sulfonates **Cs6-Cs18** according to the general description.

Sulfonate	Formula	Mass (g/mol)	Time (h)	Tem.(°C)	Yield (%)
**Cs6**	$C_{24}H_{41}O_6SCs$	590.55	24	80	39.0
**Cs8**	$C_{30}H_{53}O_6SCs$	674.71	24	80	39.6
**Cs10**	$C_{36}H_{65}O_6SCs$	758.87	24	80	49.8
**Cs12**	$C_{42}H_{77}O_6SCs$	843.04	24	80	58.5
**Cs14**	$C_{48}H_{89}O_6SCs$	927.20	24	80	54.3
**Cs16**	$C_{54}H_{101}O_6SCs$	1011.36	36	100	54.2
**Cs18**	$C_{60}H_{113}O_6SCs$	1095.52	48	100	61.4

Procedure for synthesis of cesium 3,4,5-tris(alkyloxy)benzene sulfonates Cs6-Cs18:

3,4,5-Trihydroxybenzenesulfonic acid (1.04g, 5mmol) and Cs_2CO_3 (16.3g, 50mmol) were mixed with 60mL dried DMF in a 100mL three-necked flask with a magnetic stirrer under a nitrogen atmosphere. At 80°C, 30mmol of a solution of the 1-bromoalkane in 10 mL DMF was added dropwise, followed by 0.25g KI. The reaction mixture was stirred for 24h at 80°C. After reaction, almost all solvent was removed at reduced pressure upon a rotary evaporator. 300mL of acetone was added to the residuum to form a white precipitate. The precipitate was isolated by filtration and dried in vacuum for 24h. The crude product was recrystallized 3 times from 100mL MeOH to give a white solid. After filtration and freeze-drying from 20mL benzene, a white powder was obtained.

Cesium 3,4,5-tris(hexyloxy)benzene sulfonate (Cs6): Yield: 0.65g≅39.0% of theory. M.p.: 50.0°C (by DSC). TLC (MeOH/$CHCl_3$=1/6): R_f=0.42. ^{1}HNMR (500MHz, $CDCl_3$, 20°C, TMS): 0.882(overlapped peaks, 9H, CH_3-), 1.278, 1.310, 1.439, 1.743 (broad, 24H, $CH_3(CH_2)_4$), 4.073(m, 6H, OCH_2), 7.060(s, 2H, 2,6-benzene-H). ^{13}CNMR (125MHz, $CDCl_3$, 20°C, TMS): 14.029, 22.641, 29.272, 30.334, 31.888, 69.281, 73.481, 104.824, 140.942, 152.974. IR (cm^{-1}): 3408.11, 2912.47, 2855.45, 1588.68, 1502.81, 1427.22, 1379.06, 1320.90, 1235.25, 1190.89, 1112.76, 1053.50, 1001.32, 990.43, 834.60, 728.70, 720.75, 640.49, 599.52, 590.05, 545.23. Elemental analysis calculated(%):C 48.81 H 7.00 S 5.43; found(%): C 47.87 H 7.10 S 4.54

Cesium 3,4,5-tris(octyloxy)benzene sulfonate (Cs8): Yield: 1.08g≅39.6% of theory. M.p.: 59.0°C (by DSC). TLC (MeOH/$CHCl_3$ =1/6): R_f=0.46. ^{1}HNMR (500MHz, $CDCl_3$, 20°C, TMS): 0.894(overlapped peaks, 9H, CH_3-), 1.299, 1.435, 1.723(broad, 36H, $CH_3(CH_2)_6$), 3.948(m, 6H, OCH_2), 7.058(s,

2H, 2,6-benzene-H). ^{13}CNMR (125MHz, $CDCl_3$, 20°C, TMS): 14.029, 22.648, 29.550, 30.359, 31.845, 69.356, 73.415, 104.290, 139.772, 139.982, 153.004. IR (cm^{-1}): 3417.42, 2919.71, 2851.21, 1586.87, 1498.91, 1467.02, 1421.41, 1390.33, 1315.85, 1234.24, 1183.98, 1107.62, 1046.05, 1004.34, 984.24, 829.60, 722.65, 661.94, 637.97, 600.28, 583.78, 543.20. Elemental analysis calculated(%): C 53.40 H 7.92 S 4.75; found(%): C 53.25 H 7.96 S 4.49

Cesium 3,4,5-tris(decyloxy)benzene sulfonate (Cs10): Yield: 1.98g≅49.8% of theory. M.p.: 84.1°C (by DSC); TLC (MeOH/$CHCl_3$ =1/6): R_f=0.49. ^{1}HNMR (500MHz, $CDCl_3$, 20°C, TMS): 0.895(overlapped peaks, 9H, CH_3-), 1.278, 1.314, 1.442, 1.720, 1.749 (broad, 48H, $CH_3(CH_2)_8$), 3.945, 3.969 (t, 6H, OCH_2), 7.050(s, 2H, 2,6-benzene-H), ^{13}CNMR (125MHz, $CDCl_3$, 20°C, TMS): 14.067, 22.761, 26.173, 29.511, 29.649, 29.558, 31.923, 68.031, 69.375, 104.332, 139.114, 140.673, 153.515. IR (cm^{-1}): 3417.59, 2918.77, 2850.41, 1584.41, 1499.15, 1467.09, 1422.54, 1389.82, 1317.41, 1234.02, 1202.72, 1184.35, 1110.23, 1046.55, 1003.97, 829.08, 721.77, 66303, 638.06, 600.89, 584.13, 543.45. Elemental analysis calculated(%): C 56.98 H 8.63 S 4.22; found(%): C 56.34 H 8.55 S 3.97

Cesium 3,4,5-tris(dodecyloxy)benzene sulfonate (Cs12): Yield: 2.08g≅58.5% of theory. M.p.:79.7 °C (by DSC); TLC (MeOH/$CHCl_3$=1/6): R_f=0.51. ^{1}HNMR (500MHz, $CDCl_3$, 20°C, TMS): 0.887, 0.895(overlapped peaks, 9H, CH_3-), 1.243, 1.409, 1.679, 1.849 (broad, 60H, $CH_3(CH_2)_{10}$),, 3.924, 3.951(t, 6H, OCH_2), 7.050(s, 2H, 2,6- benzene-H). ^{13}CNMR (125MHz, $CDCl_3$, 20°C, TMS): 14.044, 22.663, 26,231, 29.377, 29.649, 29.739, 30.384, 31.923, 69.384, 73.429, 104.339, 139.765, 139.811, 153.015. IR (cm^{-1}): 3424.03, 2917.67, 2849.82, 1584.04, 1499.36, 1467.09, 1422.16, 1390.68, 1317.50, 1235.68, 1182.70, 1111.02, 1047.11, 997.12, 829.21, 721.25, 663.09, 637.81, 600.27, 543.55. Elemental analysis calculated(%): C 59.84 H 9.21 S 3.80; found(%): C 59.68 H 9.12 S 3.53

Cesium 3,4,5-tris(tetradecyloxy)benzene sulfonate (Cs14): Yield: 2.52g≅54.3% of theory. M.p.: 43.0°C (by DSC); TLC (MeOH/$CHCl_3$=1/6): R_f=0.56; ^{1}HNMR (500MHz, $CDCl_3$, 20°C, TMS) : 0.885(overlapped peaks, 9H, CH_3-), 1.281, 1.319, 1.448, 1.742, 1.797(broad, 72H, $CH_3(CH_2)_{12}$), 3.976, 4.011(t, 6H, OCH_2), 7.162(s, 2H, 2,6-benzene-H). ^{13}CNMR (125MHz, $CDCl_3$, 20°C, TMS): 14.088, 22.691, 26.253, 29.397, 29.592, 29.786, 30.404, 31.946, 69.334, 73.429, 104.326, 139.707, 139.851, 152.956. IR (cm^{-1}): 3419.24, 2956.42, 2917.37, 2850.11, 1585.25, 1497.16, 1466.48, 1421.46, 1388.76, 1316.60, 1235.05, 1109.83, 1045.30, 827.97, 720.94, 659.52, 637.64, 598.50, 542.99. Elemental analysis calculated(%): C 62.18 H 9.68 S 3.46; found(%): C 61.40 H 9.41 S 3.12

Cesium 3,4,5-tris(hexadecyloxy)benzene sulfonate (Cs16): Yield: 2.74g≅54.2% of theory. M.p.: 56.9°C (by DSC). TLC (MeOH/$CHCl_3$ =1/6): R_f=0.48. ^{1}HNMR 500MHz, $CDCl_3$, 20°C, TMS): 0.900 (overlapped peaks, 9H, CH_3-), 1.280, 1.304, 1.353, 1.694, 1.84(broad, 84H, $CH_3(CH_2)_{14}$), 3.929, 3.987(overlapped peaks, 6H, OCH_2), 7.164(s, 2H, 2,6-benzene-H). ^{13}CNMR (125MHz, $CDCl_3$, 20°C, TMS): 14.089, 22.686, 26.228, 29.227, 29.484, 29.788, 30.390, 31.938, 63.956, 68.031, 73.400, 104.413, 139.600, 139.979, 152.876, 155.457. IR (cm^{-1}): 2917.49, 2849.83, 1584.86, 1466.36, 1417.36, 1379.17, 1310.74, 1274.46, 1227.86, 1195.63, 1111.26, 1045.50, 960.43, 886.40, 791.31, 720.66, 659.92, 599.18, 540.38. Elemental analysis calculated(%): C 64.13 H 10.07 S 3.17; found(%): C 63.79 H 9.83 S 2.80

Cesium 3,4,5-tris(octadecyloxy)benzene sulfonate (Cs18): Yield: 3.36g≅61.4% of theory. M.p.: 71.7°C (by DSC); TLC (MeOH/$CHCl_3$ =1/6): R_f=0.58. ^{1}HNMR (500MHz, $CDCl_3$, 20°C, TMS): 0.898(overlapped peaks, 9H, CH_3-), 1.278, 1.432, 1.697, 1.740, 1.799(broad, 96H, $CH_3(CH_2)_{16}$), 3.927, 3.999 (overlapped peaks, 6H, OCH_2), 7.159(s, 2H, 2,6-benzene-H). ^{13}CNMR 125MHz, $CDCl_3$, 20°C, TMS): 14.057, 22.657, 26.151, 29.454, 29.701, 29.737, 63.980, 69.170, 76.981, 104.681, 139.317, 140.673, 152.662, 155.439. IR (cm^{-1}): 2915.88, 2849.16, 1585.16, 1469.25, 1419.53, 1383.56, 1317.34, 1230.74, 1187.30, 1105.62, 1047.24, 839.41, 718.38, 664.76, 603.49, 543.08. Elemental analysis calculated(%): C 65.78 H 10.40 S 2.93; found(%): C 65.87 H 10.46 S 2.62

Procedure for synthesis of tetrabutylammonium 3,4,5-tris(alkyloxy)benzene sulfonates Bu6-Bu18:

In a 50mL two-necked flask with a magnetic stirrer, cesium 3,4,5-tris(alkyloxy-benzenesulfonate (1mmol) and tetrabutylammonium bromide (1.62g, 5mmol) were dissolved in 25mL CH_2Cl_2. After stirring for 1h and 10mL H_2O was added subsequently, the reaction mixture was continued stirring for 24h under a nitrogen atmosphere at room temperature. The mixture was transferred to a separation funnel, and the solvent of the collected lower layer was completely removed at reduced pressure on a rotary evaporator. 50mL H_2O was added to form a suspension. That was extracted for 3 times with 50mL benzene each, and dried for 3 times with anhydrous sodium sulfate. The further purification was done by column chromatography over silica gel with a mobile phase consisting of a mixture of MeOH and $CHCl_3$ (volume ratio=1:9) to give a white solid.

Table S4. Synthetic conditions to prepare sulfonates **Bu6-Bu18** according to the general description.

Sulfonate	Formula	Mass (g/mol)	Time (h)	Tem.(°C)	Yield (%)
**Bu6**	$C_{40}H_{77}NO_6S$	700.11	24	r. t.	40.9
**Bu8**	$C_{46}H_{89}NO_6S$	784.28	24	r. t.	50.5
**Bu10**	$C_{52}H_{101}NO_6S$	868.44	24	r. t.	52.0
**Bu12**	$C_{58}H_{113}NO_6S$	952.60	24	r. t.	48.1
**Bu14**	$C_{64}H_{125}NO_6S$	1036.76	30	r. t.	62.0
**Bu16**	$C_{70}H_{137}NO_6S$	1120.92	36	r. t.	73.2
**Bu18**	$C_{76}H_{149}NO_6S$	1205.08	45	r. t.	63.7

r.t.: room temperature.

Tetrabutylammonium 3,4,5-tris(hexyloxy)benzene sulfonate (Bu6): Yield: 284mg≅40.9% of theory, wax-like solid. M.p.: 50.0°C (by DSC). TLC (MeOH/$CHCl_3$=1/9): R_f=0.24. ^{1}HNMR (500MHz, $CDCl_3$, 20°C, TMS): 0.887, 0.986(overlapped peaks, 21H, CH_3-, $N[(CH_2)_3CH_3]_4$), 1.308, 1.411, 1.454 (overlapped peaks, 26H, $CH_3(CH_2)_3$-, $N[(CH_2)_2CH_2CH_3]_4$), 1.658, 1.763(overlapped peaks, 14H, OCH_2CH_2-, $N[CH_2CH_2CH_2CH_3]_4$), 3.282(m, 8H, $N[CH_2(CH_2)_2CH_3]_4$), 3.901, 3.977(m, 6H, OCH_2-), 7.153(s, 2H, 2,6-benzene-*H*). ^{13}CNMR (125MHz, $CDCl_3$, 20°C, TMS): 14.180, 14.261, 19.931, 22.797, 24.291, 25.927, 29.577, 29.850, 31.745, 32.112, 59.113, 69.233, 73.470, 105.131, 138.926, 142.479, 152.595. IR (cm^{-1}): 2956.11, 2922.42, 2852.71, 2338.55, 1583.75, 1493.00, 1466.46, 1420.08, 1380.99, 1314.52, 1276.64, 1223.78, 1198.05, 1104.86, 1037.36, 885.58, 842.01, 805.60, 745.48, 722.69, 654.56, 628.17, 585.77, 535.95. Elemental analysis calculated(%): C 68.62 H 11.09 N 2.00 S 4.58; found(%):C 69.24 H 11.12 N 2.18 S 4.22

Tetrabutylammonium 3,4,5-tris(octyloxy)benzene sulfonate (Bu8): Yield: 396mg≅50.5% of theory, wax-like solid. M.p.: 42.0°C (by DSC). TLC (MeOH/$CHCl_3$=1/9): R_f=0.26. ^{1}HNMR (500MHz, $CDCl_3$, 20°C, TMS): 0.876, 0.980(overlapped peaks, 21H, CH_3-, $N[(CH_2)_3CH_3]_4$), 1.268, 1.403, 1.446 (overlapped peaks, 38H, $CH_3(CH_2)_5$-, $N[(CH_2)_2CH_2CH_3]_4$), 1.650, 1.759(overlapped peaks, 14H, OCH_2CH_2-, $N[CH_2CH_2CH_2CH_3]_4$), 3.291(m, 8H, $N[CH_2(CH_2)_2CH_3]_4$), 3.896, 3.970(m, 6H, OCH_2-), 7.151(s, 2H, 2,6-benzene-*H*). ^{13}CNMR (125MHz, $CDCl_3$, 20°C, TMS): 13.831, 14.235, 19.916, 22.828, 24.262, 26.298, 29.464, 29.634, 30.482, 32.014, 59.046, 69.241, 73.478, 105.109, 138.875, 142.592, 152.575. IR (cm^{-1}): 2957.43, 2921.34, 2852.57, 1584.64, 1491.72, 1465.92, 1418.93, 1380.49, 1311.95, 1274.21, 1223.76, 1197.71, 1104.47, 1036.49, 884.47, 841.56, 805.05, 743.92, 723.97, 652.73, 627.90, 595.01, 534.85. Elemental analysis calculated(%): C 70.45 H 11.44 N 1.79 S 4.09; found(%): C 69.42 H 11.45 N 2.05 S 3.78

Tetrabutylammonium 3,4,5-tris(decyloxy)benzene sulfonate (Bu10): Yield: 452mg≅52.0% of theory, wax-like solid. M.p.: 58.3°C (by DSC). TLC (MeOH/$CHCl_3$=1/9): R_f=0.25. ^{1}HNMR (500MHz, $CDCl_3$, 20°C, TMS): 0.882, 0.992(overlapped peaks, 21H, CH_3-, $N[(CH_2)_3CH_3]_4$), 1.269, 1.417, 1.462(overlapped peaks, 50H, $CH_3(CH_2)_7$-, $N[(CH_2)_2CH_2CH_3]_4$), 1.665, 1.765(overlapped peaks, 14H, OCH_2CH_2-, $N[CH_2CH_2CH_2CH_3]_4$), 3.290(m, 8H, $N[CH_2(CH_2)_2CH_3]_4$), 3.902, 3.978(m, 6H, OCH_2-), 7.159(s, 2H, 2,6-benzene-*H*). ^{13}CNMR (125MHz, $CDCl_3$, 20°C, TMS): 13.846, 14.265, 19.933, 22.862, 24.283, 26.323, 29.604, 29.843, 30.504, 32.101, 59.081, 69.250, 73.488, 105.124, 138.941, 142.523, 152.595. IR (cm^{-1}): 2957.62, 2920.90, 2871.51, 2851.82, 1585.09, 1492.04, 1466.38, 1419.01, 1380.64, 1312.41, 1274.33, 1223.57, 1197.35, 1104.99, 1038.01, 984.93, 884.64, 839.70, 805.70, 744.54, 722.03, 653.79, 639.71, 627.68, 593.57, 542.82, 530.63. Elemental analysis calculated(%): C 71.92 H 11.72 N 1.61 S 3.69; found(%): C 72.08 H 11.86 N 1.88 S 3.52

Tetrabutylammonium 3,4,5-tris(dodecyloxy)benzene sulfonate (Bu12): Yield: 458mg≅48.1% of theory, white powder. M.p.: 12.9°C (by DSC). TLC (MeOH/$CHCl_3$=1/9): R_f=0.24. ^{1}HNMR (500MHz, $CDCl_3$, 20°C, TMS): 0.881, 0.993(overlapped peaks, 21H, CH_3-, $N[(CH_2)_3CH_3]_4$), 1.265, 1.421, 1.465(overlapped peaks, 62H, $CH_3(CH_2)_9$-, $N[(CH_2)_2CH_2CH_3]_4$), 1.645, 1.749(overlapped peaks, 14H, OCH_2CH_2-, $N[CH_2CH_2CH_2CH_3]_4$), 3.318(m, 8H, $N[CH_2(CH_2)_2CH_3]_4$), 3.901, 3.980(m, 6H, OCH_2-), 7.160(s, 2H, 2,6-benzene-*H*). ^{13}CNMR (125MHz, $CDCl_3$, 20°C, TMS): 13.810, 14.240, 19.917, 22.837, 24.284, 26.294, 29.576, 29.854, 30.464, 32.088, 59.140, 69.207, 73.443, 105.135, 138.896, 142.418, 152.568. IR (cm^{-1}): 2957.33, 2918.37, 2850.84, 1584.12, 1492.25, 1466.60, 1418.38, 1380.25, 1313.47, 1274.83, 1222.66, 1197.25, 1106.42, 1039.26, 984.44, 885.92, 842.64, 807.74, 721.66, 654.50, 639.70, 628.54, 593.61, 543.04, 530.84. Elemental analysis calculated(%): C 73.13 H 11.96 N 1.47 S 3.37; found(%): C 73.88 H 11.97 N 1.78 S 3.12

Tetrabutylammonium 3,4,5-tris(tetradecyloxy)benzene sulfonate (Bu14): Yield: 642mg≅62.0% of theory, white powder. M.p.: 33.1°C (by DSC). TLC (MeOH/$CHCl_3$=1/9): R_f=0.28. ^{1}HNMR (500MHz, $CDCl_3$, 20°C, TMS): 0.891, 0.989(overlapped peaks, 21H, CH_3-, $N[(CH_2)_3CH_3]_4$), 1.260, 1.401,

1.459(overlapped peaks, 74H, $CH_3(CH_2)_{11}$-, $N[(CH_2)_2CH_2CH_3]_4$), 1.654, 1.746(overlapped peaks, 14H, OCH_2CH_2-, $N[CH_2CH_2CH_2CH_3]_4$), 3.305(m, 8H, $N[CH_2(CH_2)_2CH_3]_4$), 3.898, 3.976(m, 6H, OCH_2-), 7.155(s, 2H, 2,6-benzene-*H*). ^{13}CNMR (125MHz, $CDCl_3$, 20°C, TMS): 13.848, 14.270, 19.942, 22.874, 24.294, 26.320, 29.627, 29.908, 30.515, 32.127, 59.107, 69.252, 73.494, 105.108, 138.923, 142.458, 152.610. IR (cm^{-1}): 2918.08, 2851.01, 1585.41, 1492.37, 1466.49, 1418.73, 1380.70, 1313.94, 1275.32, 1224.13, 1197.66, 1111.95, 1039.34, 886.66, 841.77, 806.87, 722.38, 654.96, 633.13, 595.94, 535.91. Elemental analysis calculated(%): C 74.14 H 12.15 N 1.35 S 3.09; found(%): C 74.99 H 12.24 N 1.64 S 2.87

Tetrabutylammonium 3,4,5-tris(hexadecyloxy)benzene sulfonate (Bu16): Yield: 820mg≅73.2% of theory, white powder. M.p.: 43.7°C (by DSC). TLC ($MeOH/CHCl_3$=1/9): R_f=0.27. ^{1}HNMR (500MHz, $CDCl_3$, 20°C, TMS): 0.879, 0.992(overlapped peaks, 21H, CH_3-, $N[(CH_2)_3CH_3]_4$), 1.260, 1.409, 1.463(overlapped peaks, 86H, $CH_3(CH_2)_{13}$-, $N[(CH_2)_2CH_2CH_3]_4$), 1.651, 1.763(overlapped peaks, 14H, OCH_2CH_2-, $N[CH_2CH_2CH_2CH_3]_4$), 3.311(m, 8H, $N[CH_2(CH_2)_2CH_3]_4$), 3.900, 3.99(m, 6H, OCH_2-), 7.158(s, 2H, 2,6-benzene-*H*). ^{13}CNMR (125MHz, $CDCl_3$, 20°C, TMS): 13.848, 14.274, 19.945, 22.872, 24.300, 26.341, 29.554, 29.863, 30.512, 32.123, 59.122, 69.255, 73.491, 105.163, 138.967, 142.472, 152.609. IR (cm^{-1}): 2916.89, 2850.09, 1585.34, 1492.50, 1467.11, 1418.57, 1380.08, 1313.12, 1224.34, 1197.58, 1111.50, 1041.15, 887.14, 844.38, 806.72, 720.68, 655.12, 638.63, 629.12, 593.95, 543.22, 531.03. Elemental analysis calculated(%): C 75.01 H 12.32 N 1.25 S 2.86; found(%): C 75.73 H 12.44 N 1.48 S 2.64

Tetrabutylammonium 3,4,5-tris(octadecyloxy)benzene sulfonate (Bu18): Yield: 768mg≅63.7% of theory, white powder. M.p.: 58.4°C (by DSC). TLC ($MeOH/CHCl_3$=1/9): R_f=0.31. ^{1}HNMR (500MHz, $CDCl_3$, 20°C, TMS): 0.897, 0.999(overlapped peaks, 21H, CH_3-, $N[(CH_2)_3CH_3]_4$), 1.238, 1.428, 1.471(overlapped peaks, 98H, $CH_3(CH_2)_{15}$-, $N[(CH_2)_2CH_2CH_3]_4$), 1.662, 1.767(overlapped peaks, 14H, OCH_2CH_2-, $N[CH_2CH_2CH_2CH_3]_4$), 3.325(m, 8H, $N[CH_2(CH_2)_2CH_3]_4$), 3.921, 3.997(m, 6H, OCH_2-), 7.159(s, 2H, 2,6-benzene-*H*). ^{13}CNMR (125MHz, $CDCl_3$, 20°C, TMS): 13.844, 14.263, 19.948, 22.864, 24.324, 26.329, 29.645, 29.873, 30.517, 32.115, 59.191, 69.291, 73.505, 105.037, 139.062, 142.126, 152.682. IR (cm^{-1}): 2917.24, 2850.06, 1584.73, 1467.25, 1419.35, 1380.65, 1314.01, 1224.73, 1197.52, 1106.62, 1043.53, 888.24, 820.65, 781.19, 720.48, 657.04, 639.37, 596.39, 532.33. Elemental analysis calculated(%): C 75.75 H 12.46 N 1.16 S 2.66; found(%): C 75.88 H 12.48 N 1.41 S 2.01

Techniques

An optical polarizing microscope Zeiss AXIOPLAN 2, equipped with a Mettler FP-90 hot stage, was used for detecting thermo - optical properties of the formed gels at different concentrations. Micrographs were taken using a digital Zeiss AxioCam MRC4 camera with a resolution of 4 mega-pixels in combination with Zeiss AxioVision software.

Differential scanning calorimetry measurements were performed on a Netzsch DSC 204 'Phoenix' calorimeter to examine the phase transition behaviors of gels, and reported as the maximum or minimum temperatures of the respective endo- or exothermic signals. All measurements were performed using about 10mg gels, and the corresponding solvent as references. In all cases, the heating and cooling rates were 10°C/min. Indium and cyclohexane was used as calibration standards.

A Zeiss Evo ®50 Scanning Electron Microscope was applied to analyze the surface morphologies of dried gels. The SEM samples were prepared as follows: a tiny piece of the dried gel obtained by evaporation of the solvent in air was chipped off and mounted onto a stub using carbon paint dag. Subsequently the sample was sputtered with platinum on a Q150R rotary-pumped sputter coater (Quorum Technologies) for 3~5 min.

A Zeiss EM 902 A Transmission Electron Microscope was used to analyze the morphologies of dried gels. The TEM samples are prepared as follows: a dried gel, prepared by evaporation of the solvent in air, was laid horizontally on a glass plane, and a carbon-coated copper grid was dipped onto the gel surface and pressed slightly. Then the grid covered by a ultrathin film was stained in the gas phase with ruthenium tetroxide (RuO_4) freshly developed from mixing 100mg ruthenium trichloride hydrate ($RuCl_3 \cdot xH_2O$) in a 5mL sodium hypochloride solution (NaClO, assay of 6~14wt% active Cl)[28-30]. For a favorable contrast the section was stained for 5~8 minutes before the investigation by TEM.

Gel preparation

Gels were prepared by means of the following typical procedures:

(a) Spontaneous gelation: In a 5mL test vial with a screw cap, 400 mg of a solvent / sulfonate mixture (1wt%, 2wt%, 5wt%, 10 wt% sulfonate) was heated in an oil bath until a transparent solution was obtained. Subsequently the resulting solution was allowed to cool down spontaneously to the selected investigation temperature (+20°C, -5°C, -20°C, dT/dt ~ 10 K/min), and left untouched for at least 4h. Then the vial was turned upside down to examine the presence of viscous flow or a solid gel.

(b) Quench gelation: As described with procedure (a), 400 mg of the mixture of gelator and solvent was heated until the solid was completely dissolved, then the solution was immediately quenched with liquid nitrogen to -196°C to yield a clear glass (dT/dt ~ 100 K/min). The resulting solid was slowly thawed to the investigation temperature (20°C, -5°C and -20°C) and left for at least 4h. The gelling capability was evaluated qualitatively by optical inspection of the state of the samples.

**Gelator**	**Cs6**	**Cs8**	**Cs10**	**Cs12**	**Cs14**	**Cs16**	**Cs18**	**Cs6**	**Cs8**	**Cs10**	**Cs12**	**Cs14**	**Cs16**	**Cs18**
Solvent	Spontaneous Gelation							Quench Gelation						
DMF(Dimethylformamide)	S	S	S	TG	TG	WG	WG	S	S	S	S	S	S	S
Ethanol	TG	TG	TG	TG	TG	S	S	S	S	S	S	S	S	S
Acetone	S	S	S	S	S	S	S	S	S	S	S	S	S	S
THF(Tetrahydrofuran)	S	S	S	TG	TG	WG	S	S	S	S	S	S	S	S
HEMA(2-hydroxyethyl methacrylate)	S	S	S	WG	WG	WG	WG	S	TG	WG	WG	WG	WG	WG
EGDMA(Ethyleneglycol dimethacrylate)	WG	WG	WG	WG	WG	WG	WG	WG	WG	WG	WG	WG	WG	WG
TEGDMA(Tetraethyleneglycol dimethacrylate)	WG	WG	WG	WG	S	S	WG	WG	WG	WG	WG	WG	WG	S
HMA(n-hexyl methacrylate)	WG	WG	WG	WG	WG	WG	WG	TG	S	S	S	S	WG	WG
EHMA(2-ethylhexyl metharcylate)	WG	WG	WG	WG	WG	WG	WG	TG	S	S	S	S	TG	S
Styrene	TG	TG	WG	WG	WG	TG	TG	S	S	S	S	S	S	S
Toluene	CG	CG	CG	CG	CG	CG	CG	S	S	S	S	S	S	S
Benzene	CG	CG	CG	CG	CG	CG	CG	S	S	S	S	S	S	S
n-Hexane	P	P	P	P	P	P	P	P	P	P	P	P	P	P

Gelator	Bu6	Bu8	Bu10	Bu12	Bu14	Bu16	Bu18	Bu6	Bu8	Bu10	Bu12	Bu14	Bu16	Bu18
Solvent	Spontaneous Gelation							Quench Gelation						
DMF(Dimethylformamide)	S	S	S	S	S	S	S	S	S	S	S	S	S	S
Ethanol	S	S	S	S	S	S	S	S	S	S	S	S	S	S
Acetone	S	S	S	S	S	S	S	S	S	S	S	S	S	S
THF(Tetrahydrofuran)	S	S	S	S	S	S	S	S	S	S	S	S	S	S
HEMA(2-hydroxyethyl methacrylate)	S	S	S	S	S	S	S	S	S	S	S	S	S	S
EGDMA(Ethyleneglycol dimethacrylate)	S	S	S	S	S	S	S	S	S	S	S	S	S	S
TEGDMA(Tetraethyleneglycol dimethacrylate)	S	S	S	S	S	S	S	S	S	S	S	S	S	S
HMA(n-hexyl methacrylate)	S	S	S	S	S	S	S	S	S	S	S	S	S	S
EHMA(2-ethylhexyl metharcylate)	S	S	S	S	S	S	S	S	S	S	S	S	S	S
Styrene	S	S	S	S	S	S	S	S	S	S	S	S	S	S
Toluene	S	S	S	S	S	S	S	S	S	S	S	S	S	S
Benzene	S	S	S	S	S	S	S	S	S	S	S	S	S	S
n-Hexane	S	S	S	S	S	S	S	S	S	S	S	S	S	S

Disclaimer/Publisher's Note: The statements, opinions and data contained in all publications are solely those of the individual author(s) and contributor(s) and not of MDPI and/or the editor(s). MDPI and/or the editor(s) disclaim responsibility for any injury to people or property resulting from any ideas, methods, instructions or products referred to in the content.
